# Tertiary lymphoid structure‐related RNA indicator as metastasis risk factor in nasopharyngeal carcinoma

**DOI:** 10.1002/ctm2.70539

**Published:** 2025-12-15

**Authors:** Zhaozheng Hou, Ping Feng, Chi‐Leung Chiang, Kazi Anisha Islam, Songran Liu, Ying Wang, Yingpei Zhang, Michael King‐Yung Chung, Ngar‐Woon Kam, Zilu Huang, Victor Ho‐Fun Lee, Anne Wing‐Mui Lee, Dora Lai‐Wan Kwong, Wai Tong Ng, Jason Wing Hon Wong, Yunfei Xia, Wei Dai

**Affiliations:** ^1^ Department of Clinical Oncology University of Hong Kong Hong Kong (SAR) PR China; ^2^ Department of Radiation Oncology Sun Yat‐Sen University Cancer Centre Guangzhou PR China; ^3^ State Key Laboratory of Oncology in South China Collaborative Innovation Center for Cancer Medicine Guangdong Key Laboratory of Nasopharyngeal Carcinoma Diagnosis and Therapy Sun Yat‐Sen University Cancer Center Guangzhou PR China; ^4^ Department of Pathology Sun Yat‐Sen University Cancer Centre Guangzhou PR China; ^5^ Laboratory for Synthetic Chemistry and Chemical Biology Hong Kong (SAR) PR China; ^6^ School of Biomedical Sciences University of Hong Kong Hong Kong (SAR) PR China; ^7^ Department of Clinical Oncology Shenzhen Key Laboratory for Cancer Metastasis and Personalized Therapy The University of Hong Kong‐Shenzhen Hospital Shenzhen PR China

**Keywords:** distant metastasis, nasopharyngeal carcinoma, Pareto optimisation, risk score, RNA sequencing

## Abstract

**Background:**

Nasopharyngeal carcinoma (NPC) patients who develop distant metastasis have significantly reduced survival rates. Therefore, understanding of metastasis and identifying high‐risk patients are important, and a robust predictive model for accurately assessing the distant‐metastasis risk before treatment is needed for personalised treatment.

**Methods:**

NPC patients diagnosed at four Hong Kong public hospitals and at Sun Yat‐Sen University Cancer Center in Guangzhou were selected. Patients were divided into two training cohorts (n = 77 and 30, respectively) and one testing cohort (n = 70). Two independent NPC cohorts collected from Sun Yat‐Sen University Cancer Center (n = 88), and a randomised phase III trial (NPC‐0501) in Hong Kong (n = 81) were used for external validation of the model‐based risk prediction.

**Results:**

Our RNA‐based risk score could stratify the patient groups into high and low risk of metastasis and disease progression in two independent external validation cohorts. In predicting NPC 3‐year distant metastasis, the score significantly improved the area under the curve from 84.8% to 90.4% when combined with the known prognostic clinical parameters. This RNA‐based risk score was highly associated with dysregulated functions of B cells and T helper 17 cells and reduced plasma B cells and tertiary lymphoid structure (TLS) formation. The analysis of biopsy samples revealed a significant enrichment of the TLS in non‐metastatic NPC patients.

**Conclusions:**

This study improved the accuracy of NPC metastasis prediction and highlight the potential association of TLS against metastatic NPC, encouraging future studies to understand how TLS interacts with NPC to prevent distant metastasis. Furthermore, the multi‐cohort Pareto‐optimisation‐based feature selection approach offers a practical method to explicitly avoid model overfitting and achieve a more robust model.

**Novelty and Impact:**

In this multicentre study, we established a new and robust predictive model for NPC distant metastasis using markers selected by a Pareto optimisation approach designed for multi‐cohort data. When combined with clinical parameters, our RNA‐based risk score significantly improved the area under the curve to 90.4%. This study revealed that reduced B‐cell immunity, and TLS formation, may be associated with NPC metastasis, providing insights for the future studies in NPC metastasis.

## Graphical abstract

We established a robust predictive model for nasopharyngeal carcinoma (NPC) distant metastasis using RNA markers. When combined with clinical parameters, our RNA‐based risk score significantly improved the area under the curve to 90.4%. This study revealed that tertiary lymphoid structure formation may be associated with lower NPC metastasis risk after first‐line treatment.

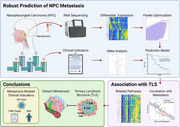

## 1 INTRODUCTION

With advancements in the treatment of nasopharyngeal carcinoma (NPC), the 5‐year relative survival rate has the potential to exceed 90%.^1^, ^2^ However, patients who develop distant metastases (cancer that spreads to distant organs after treatment) have a significantly reduced survival rate.^3^, ^4^ Therefore, further understanding of NPC metastasis and identifying patients likely to develop distant metastasis after primary treatment are important, and a robust predictive model for accurately assessing the risk of distant metastasis in patients before treatment is needed to implement personalised medical plans.^5^ A previous study from Chen et al.^6^ identified multiple gene markers for different NPC tumour subtypes and proposed the cell cycling score that had significant association with survival of NPC. Similar predictive models based on gene panels will benefit molecular subtyping and enhance the understanding of the mechanisms related to NPC development. Furthermore, Tang et al.^7^ and Liu et al.^8^ presented a 13‐gene and a four‐gene metastasis‐prediction model for NPC based on single cohort data. However, the detailed mechanisms underlying the selected genes remained to be investigated.

Moreover, a substantial challenge inherent in multicentre studies is controlling the effects of site‐specific factors when utilising their data to create robust and generalisable models.^9^ Notwithstanding the remarkable progress made in standardising data collection and processing over recent decades, the myriad of potential sources of bias across data resources continues to exert a non‐negligible influence on current multicentre studies.^10^, ^11^ Despite the development of numerous packages to address the impact of batch and cohort differences,^12^, ^13^, ^14^, ^15^ some correction methods have been shown to enhance model performance significantly. However, in clinical practice, it is common that the expected batch effect correction method cannot be applied to patient data because of sample size and/or violation of certain assumptions of the method, which is a serious concern for this type of study.

In this vein, this study aimed to identify biomarkers through RNA sequencing (RNA‐seq) for primary NPC to develop a corresponding prediction model incorporating clinical data for distant metastasis, and to facilitate patient stratification before primary treatment. Pareto optimisation has been employed to reconcile marker selection based on multiple cohorts.^16^, ^17^ Rather than attempting to completely eradicate site‐specific effects between cohorts and construct a model based on corrected data, we aimed to enhance our model's performance on new cohorts while acknowledging discrepancies between cohorts.^18^ This methodology circumvents the need for cross‐cohort normalisation to allow for explicit visualisation of the balance between cohorts. It ensures that the selected features and corresponding predictive model remain robust and accurate across cohorts, even when there are insufficient samples for effective batch‐effect correction.

## 2 METHODS

### 2.1 Study workflow

This study classified the NPC patients with distant metastasis‐free survival (DMFS) under 3 years as early metastatic NPC (MET‐NPC), whereas the NPC patients with DMFS over 3 years or without distant metastasis 5 years after treatment as non‐metastatic NPC (non‐MET‐NPC). Three‐year DMFS was used as the cutoff, as previous studies have reported that over 70% of distant metastases occurred in this period.^19^

The model established in this study was based on three cohorts: training cohort 1, training cohort 2 and the testing cohort. Training cohort 1 (*n* = 77), comprised of 39 MET‐NPC and 38 non‐MET‐NPC cases, was recruited from four Hong Kong hospitals (Queen Mary, Queen Elizabeth, Princess Margaret and Pamela Youde Nethersole Eastern Hospitals); training cohort 2 (*n* = 30), consisting of 13 MET and 17 non‐MET cases and the testing cohort (*n* = 70), with 23 MET and 47 non‐MET cases, were both recruited from Sun Yat‐Sen University Cancer Center in Guangzhou.

Two external independent cohorts were used to validate the model for risk prediction: validation cohort 1 (*n* = 88) from the Sun Yat‐Sen University Cancer Center in Guangzhou, with 88 valid cases,^20^ and validation cohort 2 (*n* = 81) from one NPC clinical trial (NPC‐0501; NCT00379262), with 81 valid cases.^21^

The clinical parameters of the training and testing cohorts are summarised in Table S1. The significance of the association of these parameters with metastasis was examined using appropriate statistical tests according to the data type and statistical conditions.^22^, ^23^ The clinical characteristics considered included relapse conditions, age at diagnosis, year of diagnosis, sex and TNM stages according to American Joint Committee on Cancer (AJCC) staging system version 8.^24^ Fisher's exact test showed a significant variation in the proportion of patients with MET‐NPC between the relapse/non‐relapse patient sample groups in training cohort 1. Tests for other characteristics (including age, sex and TNM staging) did not reveal any significant associations.

**FIGURE 1:**
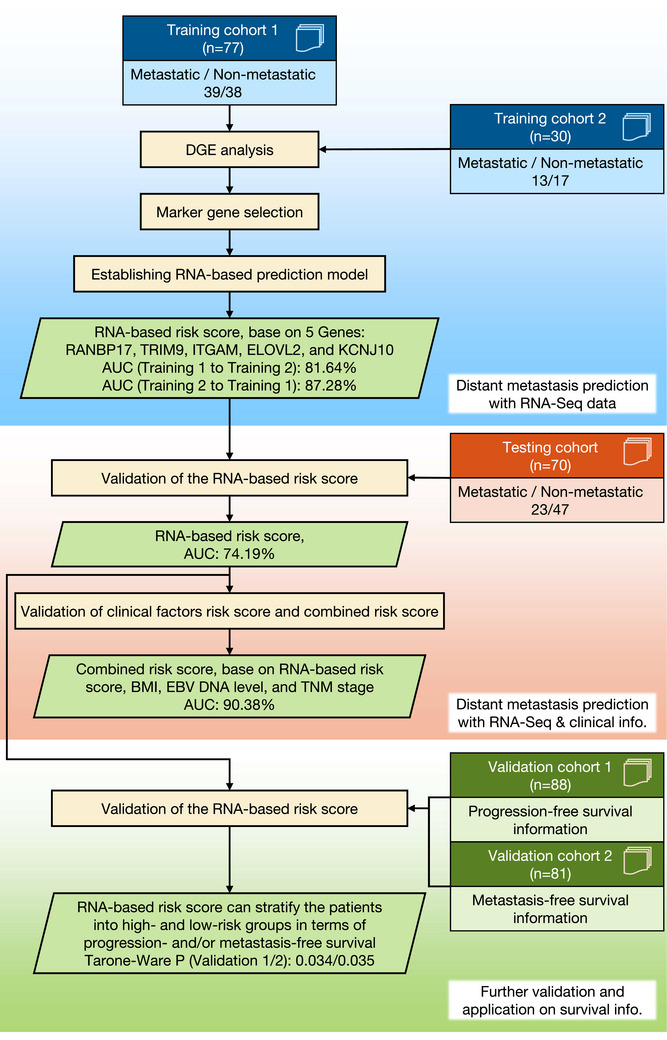
Study workflow. Differential gene expression (DGE) analysis was conducted on training cohorts 1 and 2 to identify common marker genes for distant metastasis. Subsequently, Pareto optimisation was utilised for further selection of robust marker combinations. Testing cohort was used to evaluate the performance of the RNA‐based model (i.e., RNA‐based risk score), and the predictions based on both clinic data only (clinical factors risk score) and the combined risk score. Validation cohorts 1 and 2 have the RNA sequencing and progression/metastasis‐free survival information, which are used to validate the model prediction as a basis for stratification of patients with high and low risk of metastasis.

Training cohorts 1 and 2 were chosen for gene marker selection and RNA‐seq‐based prediction model (RNA‐based risk score). Subsequently, the testing cohort was used to evaluate the performance of the RNA‐based risk score and to determine whether it could enhance the accuracy of the predictions generated using clinical information. Finally, the two external validation cohorts were used to examine whether the proposed RNA‐based risk score could identify the high‐risk patients with worse progression‐free and/or metastasis‐free survival (Figure 1).

### 2.2 Patient recruitment and study approval

Patients diagnosed with NPC at four Hong Kong hospitals (Queen Mary, Queen Elizabeth, Princess Margaret and Pamela Youde Nethersole Eastern Hospitals) between 2010 and 2018 (training 1 cohort), or at Sun Yat‐Sen University Cancer Center in Guangzhou between 2011 and 2021 (training 2 and testing cohorts) were selected if they fulfilled the following criteria: were pathologically diagnosed with undifferentiated non‐keratinising NPC, had prospective clinical data, and were available for primary nasopharyngeal foci sample collection. All cases were treated according to National Comprehensive Cancer Network guidelines and were monitored through medical visits and telephone interviews. Cancer progression was confirmed by histopathological examination or by at least two other diagnostic tests, including cytopathologic tests, computed tomography, magnetic resonance imaging, positron emission tomography, single‐photon emission computed tomography, ultrasound examination and endoscopy. All participants in the training 1 cohort were followed up until 31 May 2023, while all participants in the training 2 and testing cohorts were followed up until 1 December 2021. Disease progression detected in distant organs after treatment was diagnosed as distant metastasis, and the cases that developed distant metastasis within 3 years were considered as early MET cases. Disease progression detected locally after CR was diagnosed as local relapse. In this study, sex was used as a biological variable. Patients were grouped according to their sex chromosomes, with males assigned the designation XY and females XX. Summary of clinical characteristics (also include age ranges of each cohort) is presented in Table S1.

Samples of validation 1 cohort were collected from Sun Yat‐Sen University Cancer Center in Guangzhou before 2017.^20^ The samples came from formalin‐fixed paraffin‐embedded (FFPE) samples taken before receive any treatment, and ensured the selected cases contains more than 50% of tumour cells. Patents of validation 2 cohort were FFPE samples recruited in Hong Kong in a randomised phase III trial (NPC‐0501; NCT00379262).^21^ Both the validation cohorts have been subject to follow‐up over a period of at least 5 years.

### 2.3 Power analysis of the study cohorts

Power analysis was performed using R package pwr (RRID:SCR_025480, v1.3‐0). For training cohort 1 (the main cohort for feature gene selection and model fitting), the power corresponding to large effect size (Cohen's *d* = .8) with a significance level of .025 is 88.7%, and with a significance level of .2 is 98.6%. We therefore used the following cutoff for this cohort: adjusted *p*‐value (false discovery rate) <.2 and *p* < .025. For the testing cohort, the power corresponding to large effect size (Cohen's *d* = .8) with a significance level of .05 is 87.3%.

### 2.4 Clinical sample preparation

Samples from training 1 and 2 cohorts and testing cohort were obtained from fresh frozen samples from biopsies before first‐line treatment. RNA was extracted from the clinical samples using the All‐Prep DNA/RNA Kit (Qiagen). The purity and integrity of RNA were examined with a Qubit and Bioanalyser, respectively. The pair‐end RNA libraries were prepared and sequenced by Novogene, following the manufacturer's protocol.

### 2.5 RNA sequencing experiments and data processing

The quality of the raw reads was examined using FastQC (RRID:SCR_014583, v0.11.8). RSeQC^25^ (RRID:SCR_005275, v2.6.4) and Picard (RRID:SCR_006525, v2.17.4) were used to investigate the alignment quality and the strand‐specificity of the RNA‐seq libraries. All samples had a mapping rate of at least 95%. STAR^26^ (RRID:SCR_005622, v2.7.5c) was utilised to align the reads onto the reference genome, which integrated the human genome (hg38) and the EBV genome (NC_007065), using default parameters. The genome annotation files for the human genome and EBV genome were downloaded from GENCODE^27^ (RRID:SCR_014966, v27) and GeneBank, respectively. HTSeq^28^ (RRID:SCR_005514, v0.9.1) was utilised to quantify read counts at the gene level in intersection‐non‐empty mode. Library normalisation and differential gene expression analysis were performed using DESeq2^29^ (RRID:SCR_000154, v1.30.1). Variance‐stabilising transformation was used to normalise counts for accounting library size differences and visualisation.

### 2.6 Pareto optimisation for feature selection

Feature selection procedure in this study involves establishing a robust linear predictive model from the 29 common gene signatures, which entails selecting a gene combination from 2^29^ ‒1 (over 536 million) feasible options, using the two independent training cohorts as a reference. Thus, it is essential to reduce the number of candidate combinations for validation in the testing cohort. Although several widely used feature selection approaches exist for single data set,^30^, ^31^, ^32^ almost all these methods require cohort merging with batch effect correction when establishing models based on multiple cohorts. To achieve a robust feature selection with balanced consideration of the two training cohorts, a cross‐disciplinary approach called Pareto optimisation^33^ has been adopted.

Pareto optimisation, also referred to as multicriteria^34^ or multi‐objective optimisation,^35^ is a widely utilised approach for identifying feasible solutions that balance multiple objectives for optimisation. Previous studies have provided examples of similar applications (for balancing multiple objectives on a single or merged cohorts).^17^, ^36^ This method is particularly useful for establishing predictive models from multiple cohorts, as it provides an approach that considers different cohorts as individual data sets in a balanced way, without attempting to merge the cohorts with batch effect correction, a challenging task especially for small‐size cohorts.^37^, ^38^ A robust marker combination should lead to an acceptable validation area under the curve (AUC) for independent testing cohorts while not being sensitive to which cohorts are selected for training or validation.

Related work was carried out with MATLAB (RRID:SCR_001622, 2024b). We defined two objectives: the AUC score with training cohort 1 as the reference for model parameter training and training cohort 2 for validation, and the performance after swapping the roles of the training cohorts. The selected gene combinations constituted a Pareto set that cannot be outperformed by other gene combinations in both objectives. A depth‐first search (traversing search) was conducted for one/three/five gene combinations, while a discrete genetic algorithm was employed for 1–10/10–15/15–20 gene combinations. To ensure the generation of sufficient valid search points during the genetic algorithm search, the gene number was set as a range rather than one value.^39^ Each gene number setting corresponded to a Pareto set of 5‒15 feasible options, a significant reduction from the 2^29^ ‒ 1 possible combinations. Subsequently, the marker combinations were validated in the testing cohort to finalise the selection of the optimal gene combination.

### 2.7 Pathway activity analysis

GSVA (RRID:SCR_021058, v1.50.1) was employed to generate Gene Ontology Biological Processes and Molecular Function (GOBP/GOMF, RRID:SCR_002811) pathway activity matrices by performing GSVA on gene expression data. To reduce the number of pathways while ensuring that metastasis‐specific pathways were retained, the Mann‒Whitney *U*‐test was applied to each pathway in the training dataset using metastasis as the outcome. A total of 2356 pathways with a *p*‐value less than .05 were kept for further analysis.

### 2.8 Deconvolution analysis

In this study, the proportion of different cell types in NPC patients was estimated according to the bulk RNA‐seq data. To establish the reference pattern of each cell type, single‐cell sequencing data from three previous studies^6^, ^40^, ^41^ on NPC primary tumours are adopted to establish the reference pattern of each cell type. The three data sets were merged with batch effect removal process using the R package Harmony (RRID:SCR_023543, v1.2.0), with the major cell types identified including tumour cells, fibroblasts, dendritic cells, myeloid cells, T cells, NK‐T, mast B, plasma B, memory B and naïve B. Doublets and proliferating cells are identified and removed in R with Seurat (RRID:SCR_016341, v5.0.3). The selection of reference genes was conducted following the methods previously established by other researchers.^42^, ^43^ For each cell type, the genes were sorted based on the *p*‐values derived from a two‐sided *t*‐test of their expression levels within that cell type compared to other cell types, with the top G genes from this initial sorting being included in the final reference for deconvolution. As demonstrated in the previous study,^42^ the value of *G* was selected to minimise the condition number with the minimum number of genes, and thus the *G* was decided to be 1509 in this study (Figure S5).

Contrary to most deconvolution analysis approaches that only consider the estimated expression level for each cell type, we also incorporated the variance in the expression level for different cell types. The overall variance of each feasible candidate gene would be computed during the search for the best‐fitting mixture. Genes with larger variances will have reduced penalties for discrepancies between estimation and observation, resulting in a superior fit to the observed data, which was also more consistent with related practical experience in multi‐omics and image‐based results. The primary deconvolution procedure employed in this study can be mathematically expressed as follows:

$$\min_{P_{1},P_{2},\cdots ,P_{n}}\sum_{i=1}^{m}\frac{{\left[\mathrm{Bulk}\;\mathrm{RN}A_{i}\times \mathrm{Scaling}\;\mathrm{factor}-\sum_{j=1}^{n}\left(P_{j}\times {\mathrm{Estimated}\;\mathrm{expression}}_{i}^{j}\right)\right]}^{2}}{\sum_{j=1}^{n}\left(P_{j}\times {\mathrm{Variance}\;\mathrm{level}}_{i}^{j}\right)}$$

where there are n cell types and m genes to consider, and $\;P_{1},P_{2},\cdots ,P_{n}$ are proportions for each cell type with $\;P_{j}\geq 0,\;\;\sum_{j=1}^{n}P_{j}=P_{1}+P_{2}+\cdots +P_{n}=1$. $\mathrm{Bulk}\;\mathrm{RN}A_{i}$ is the bulk RNA‐seq normalised count for the gene with index i, ${\mathrm{Estimated}\;\mathrm{expression}}_{i}^{j}$ is the averaged expression level of gene i for cell type j and ${\mathrm{Variance}\;\mathrm{level}}_{i}^{j}$ is the expression level variance of gene i for cell type j. The MATLAB function particleswarm was employed to solve this mathematical formula.

In the comparison with typical deconvolution tools that does not bring expression variance into consideration, such as CIBERSORT (Figure S8), the two proportion estimation results for primary cell types exhibit a significant correlation. However, the proposing ‘expression and variance’ approach yields less unreasonably extreme results, such as proportions of zero and unrealistically high proportions.

### 2.9 Principal component analysis of single‐cell data

The three single‐cell data sets^6^, ^40^, ^41^ used for deconvolution were also used to investigate the role of RNA‐based risk score at the single‐cell level. The first three principal components were computed after merging the three data sets using the R package Harmony (RRID:SCR_023543, v1.2.0) and quantified in the format of log_10_(normalised read count + 1), which is the same expression level format for the RNA‐based risk score. Furthermore, the PC projections of the risk score and associated pathways were calculated. These projections indicate the major varying direction of the risk score and pathway activities in the principal component analysis (PCA) plot. It needs to pointed out that both the risk score and the pathways involve more than three genes, so the projection vectors only provide the main directions of variation, and the values could vary non‐linearly perpendicular to the projection vector.

### 2.10 Multiplex immunofluorescence staining

For 12 non‐MET and 20 MET cases from an independent HK cohort, 5‐µm FFPE tissue sections were fluorescently stained with DNA dye (SYTO 13) and the primary antibodies targeting CD20 (NanoString Technologies, clone ID: IGEL/773, RRID:AB_3308987), CD45 (NanoString Technologies, clone ID: D9M8I, RRID:AB_2922785), PanCK (NanoString Technologies, clone ID: AE‐1/AE‐3, RRID:AB_2249764), as well as the secondary antibody (ThermoFisher Goat Anti‐rabbit, A‐11012, RRID:AB_2534079). The stained slides were imaged on the GeoMx DSP platform. The total number of tertiary lymphatic structures (TLSs) per slide, which was defined as the aggregate of more than 100 CD20+ CD45+ PanCK‒ cells surrounded by CD20‒ CD45+ PanCK‒ cells, was normalised by the tissue area presented on the whole‐slide image.^44^ The two‐sided Student's *t*‐test was used to evaluate significant difference in TLS numbers between the non‐MET and the MET groups.

The TLS enrichment is quantified by the number of identified TLS per square millimeter for each biopsy sample:

$$\mathrm{TLS}\;\;\left(mm^{2}\right)=\frac{\mathrm{Number}\;\mathrm{of}\;\mathrm{TLS}\;\mathrm{identified}}{\mathrm{Area}\;\mathrm{of}\;\mathrm{valid}\;\mathrm{stained}\;\mathrm{cells}}$$

where regions of valid stained cells are defined by areas with strong staining signals (signal intensity significantly higher than the background in at least one channel) and high‐quality structural details of cells. Structural similarity index (SSIM)^45^ was used to quantify structural detail quality, and pixels with SSIM <.95 compared to a low‐resolution version of the same image (10% PPI) were considered low‐quality.

### 2.11 Statistical analysis

Unless otherwise noted, all significant levels of continuous variables concerning categorical grouping factors (such as metastasis status) were calculated using the Wilcoxon rank‐sum test.

The dendrogram heat maps were generated using MATLAB R2024b, and the element order was optimised by using the optimalleaforder function with the Criteria type set to ‘group’.^46^

The statistical process for the forest plot was processed using MATLAB (RRID:SCR_001622) and SPSS (RRID:SCR_002865). The optimal operating point calculated with the MATLAB perfcurve function was chosen as the cutoff for each clinical parameter,^47^, ^48^, ^49^ and the odds ratio and *p‐*values were computed in SPSS (RRID:SCR_002865, v26.0).

For the typical feature selection approach with LASSO, the calculation was performed in MATLAB with the built‐in Statistics and Machine Learning Toolbox.^50^, ^51^ Batch effect correction was performed using Limma ((RRID:SCR_010943, v3.52.4).^12^

Spearman's correlation analysis was used to quantify the correlations between the identified predictive factors. The correlation‐directed graph was then generated using MATLAB's graph and layout functions (RRID:SCR_001622), using the significance level *p*‐value on the log scale as the factor of attraction,^52^ to plot factors with a lower *p‐*value closer together. Wilcoxon rank‐sum test was used to calculate the significance of factor differences between MET‐NPC and non‐MET‐NPC groups to decide the size and color of each factor on the graph.

## 3 RESULTS

### 3.1 Feature selection and RNA‐based model construction

**FIGURE 2:**
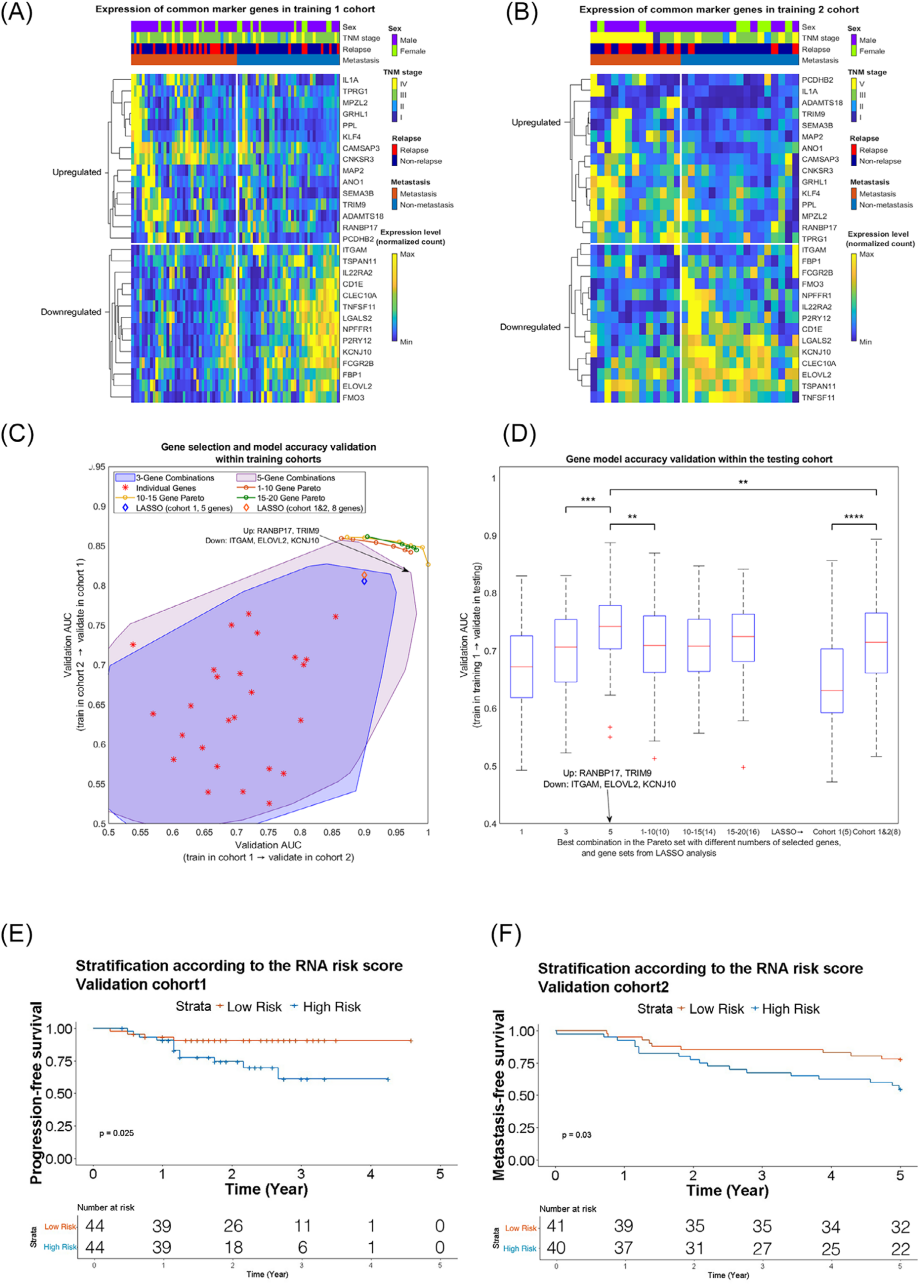
Differential gene expression analysis on the two training cohorts, and Pareto optimisation. The figure depicts the expression levels of these marker genes in the training 1 (*n* = 77, A) and training 2 (*n* = 30, B) cohorts, grouped by metastasis status and enrichment direction. Twenty‐nine common marker genes were identified in metastatic and non‐metastatic nasopharyngeal carcinoma. The Pareto frontiers (top‐right border for each gene‐number‐group; C) were validated within the testing cohort. The performance of these Pareto‐optimised gene combinations is shown in (D). The area under the curve (AUC) score of the five‐gene combination was significantly better than the combinations with fewer or more genes. Survival analysis on validation 1 cohort shows that the RNA score can effectively identify the high‐risk group (log‐rank *p* = .025, Tarone‒Ware *p* = .034) (E). The plot *F* base on validation 2 cohort shows that the metastasis‐free survival information and the RNA score can identify the high‐risk group with log‐rank *p* = .030, Tarone‒Ware *p* = .035).

A total of 29 common genes (15 upregulated and 14 downregulated genes, Figure 2) were differentially expressed in MET‐NPC when compared to non‐MET‐NPC, using training 1 after adjustment of relapse condition between MET‐NPC and non‐MET‐NPC (77 patients, absolute log2 fold‐change > .5, adjusted *p* < .2 and *p* < .025) and training 2 (30 patients, absolute log2 fold‐change > .5 and *p* < .05) cohorts. The cutoff for significance was determined by the power analysis of the study cohorts.

A cross‐disciplinary approach, known as Pareto optimisation,^33^ was adopted to establish a robust feature selection from the identified 29 genes (see Section 2.6). In brief, this method evaluates marker gene subsets with the AUC scores, as using one of the training cohorts as the reference to establish a linear prediction model and validate its accuracy in the other training cohort. With two training cohorts, two AUC scores could be obtained for each feature selection, and an optimal robust selection is expected to exhibit two higher AUC scores compared to sub‐optimal feature selections. In practice, it is extremely unlikely to find a ‘perfect’ selection that outcompetes every other selection in both AUC scores. A series of candidates (which cannot outcompete any other in both scores at the same time) would form a ‘Pareto frontier’ (Figure 2C).

To generate the Pareto frontiers, all possible gene combinations of one, three or five genes was evaluated (the achievable regions of the AUC scores, also known as feasible regions, are shown in Figure 2C). For combinations of three or five genes, the Pareto frontier corresponds to the top‐right parts of the border of the feasible objective regions. A discrete genetic algorithm search was conducted to identify the optimal combinations with 1–10, 10–15 or 15–20 genes from a large number of feasible candidates (Figure 2C).

Subsequently, the combinations that emerged from the Pareto optimal set were validated using the independent testing cohort (Figure 2D). Interestingly, the performance of models comprising more than ten genes in the testing cohort was not significantly improved when compared to the five‐gene model (*p* < .01), regardless of their better AUC scores in the previous step (Figure 2C). This indicates that the inclusion of more factors does not necessarily lead to increased accuracy, as the extra factors may contain duplicated information, whereas overfitting is more likely to occur with more factors included. We also applied LASSO, a standard method for feature selection, using both training cohorts and training cohort 1 alone (with batch effect correction by Limma^53^, ^54^), the Pareto approach performed better than LASSO when validated on both training cohorts and the testing cohort (*p* < .0001, Figure 2D).

The final model was established using the combination of the five genes *RANBP17*, *TRIM9*, *ITGAM*, *ELOVL2* and *KCNJ10*, and its validation AUC in the testing cohort was 74.2% (Figure 2D). The corresponding score formula (obtained from training cohort 1) was as follows:

$$\begin{array}{ccc} \mathrm{RiskScor}e_{\mathrm{RNA}} & & =.593\times RANBP17+.710\times TRIM9\\ & & -.903\times ITGAM-.803\times ELOVL2\\ & & -.815\times \mathrm{KCNJ}10+3.190 \end{array}$$

The expression of each gene was calculated as log_10_(normalised read count + 1). The average risk score was set to be 0 for non‐MET samples and 1 for the MET samples. The operating point (cutoff)^47^ of this score is .507.

In the external validation with survival outcomes, the RNA score could effectively identify the high‐risk group with shorter disease progression‐free survival in validation cohort 1 (log‐rank *p *= .025, Tarone‒Ware *p *= .034, Figure 2E) and shorter metastasis‐free survival in the validation cohort 2 (log‐rank *p* = .030, Tarone‒Ware *p *= .035, Figure 2F).

### 3.2 Clinical parameters associated with distant metastasis

**FIGURE 3:**
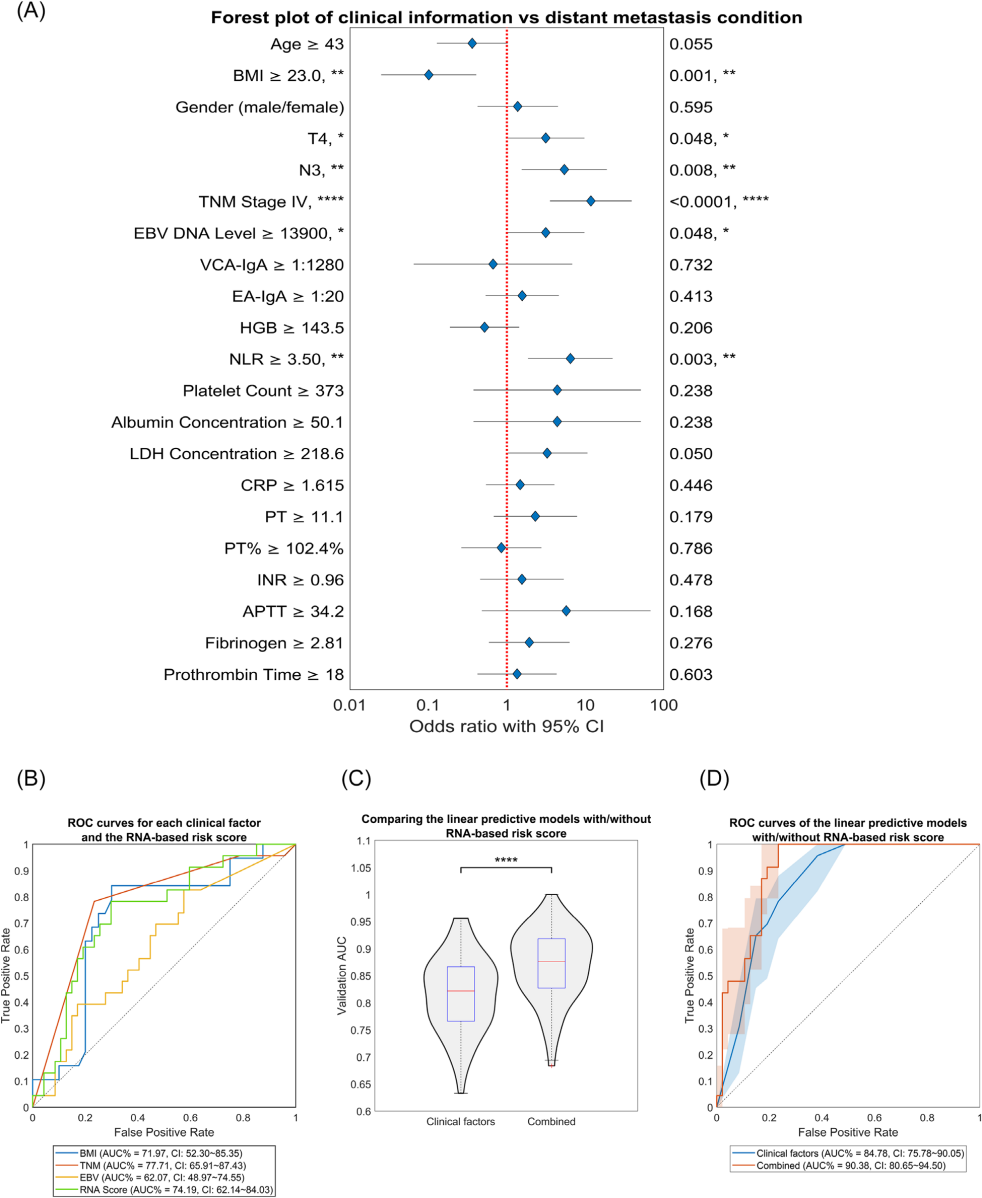
Proposed RNA‐based risk score demonstrated a notable enhancement in prediction accuracy when added to a clinical data‐based model. (A) Forest plot on the right illustrates the results. The optimal operating points of the corresponding receiver operating characteristic (ROC) curves serve as the cutoff points for each variable. Results of the correlation and multi‐variable model analyses are presented in Figure S2 and S3 and Table S1. (B) Receiver operating characteristic (ROC) of the clinical factors and RNA‐based risk score. (C) Comparison of prediction performance between the clinical feature‐only model and the combined model with clinical parameters and RNA‐based risk score. When applying the Mann‒Whitney *U*‐test to bootstrapping replicates, the model incorporating an RNA‐based score attained a significantly higher validation area under the curve (AUC) (*p* < .0001). (D) ROC curves for the clinical model and a combined model with clinical parameters and RNA‐based risk score.

To obtain a predictive model incorporating clinical information with bulk RNA‐seq data and ascertain the extent of accuracy enhancement by incorporating the five‐gene RNA risk score, we initially conducted an exhaustive examination of the 25 clinical characteristics available for analysis in the testing cohort. Body mass index (BMI), T stage, N stage, TNM stage (AJCC version 8), plasma EBV DNA level (Epstein‒Barr virus) and neutrophil‐to‐lymphocyte ratio were significantly associated with metastasis (Figure 3 and Table S2). Correlations of the clinical parameters were evaluated using Spearman's correlation analysis (Figures S2 and S3). In the multivariable analysis, BMI and TNM stage showed independent effects on metastasis (*p* < .05, Table S3). Given that plasma EBV DNA level has been reported and adopted clinically as a principal factor associated with distant metastasis,^55^ we also incorporated EBV DNA level into the prediction model. Ultimately, three clinical characteristics, TNM stage (version 8), BMI and EBV DNA level were utilised to construct a linear clinical‐information‐based model (Figure 3).

### 3.3 Construction of the prediction model combined clinical parameters and RNA‐based risk score

To construct a combined prediction model incorporating clinical factors and RNA‐based risk scores, and to validate the performance of this combined model, the samples in the testing cohort were randomly split into two groups: 70% for training (model parameter calculation) and 30% for validation (risk score AUC calculation). This procedure was repeated 200 times to ensure the robustness of results. As illustrated in Figure 3, adding the RNA‐based risk score resulted in a notable enhancement of the AUC (*p* < .0001). The average integrated discrimination improvement for the RNA risk score across 200 bootstraps was 10.0% (*p* < .0001). The formula for the risk score incorporating clinical data with RNA expression was as follows:

$$\begin{array}{ccc} \mathrm{RiskScor}e_{\mathrm{Clinical}\&\mathrm{RNA}} & & =-.610\times \mathrm{BM}I_{\geq 23}+.042\\ & & \times \mathrm{EB}V_{\geq 4000}+.873\times \mathrm{TN}M_{=4}+.405\\ & & \times \mathrm{RiskScor}e_{\mathrm{RNA}}+.388 \end{array}$$

The model was derived from average model parameters estimated from 200 bootstrapping replicates in the testing cohort. The average risk score was 0 for the non‐MET samples and 1 for the MET samples. The operating point (cutoff)^47^ of this score is .714. The risk score does not show significant correlations with other factors, including relapse condition, age and gender in any training cohorts or the testing cohort (Table S6).

### 3.4 RNA‐based risk scores are negatively correlated with active immune cell functions

**FIGURE 4:**
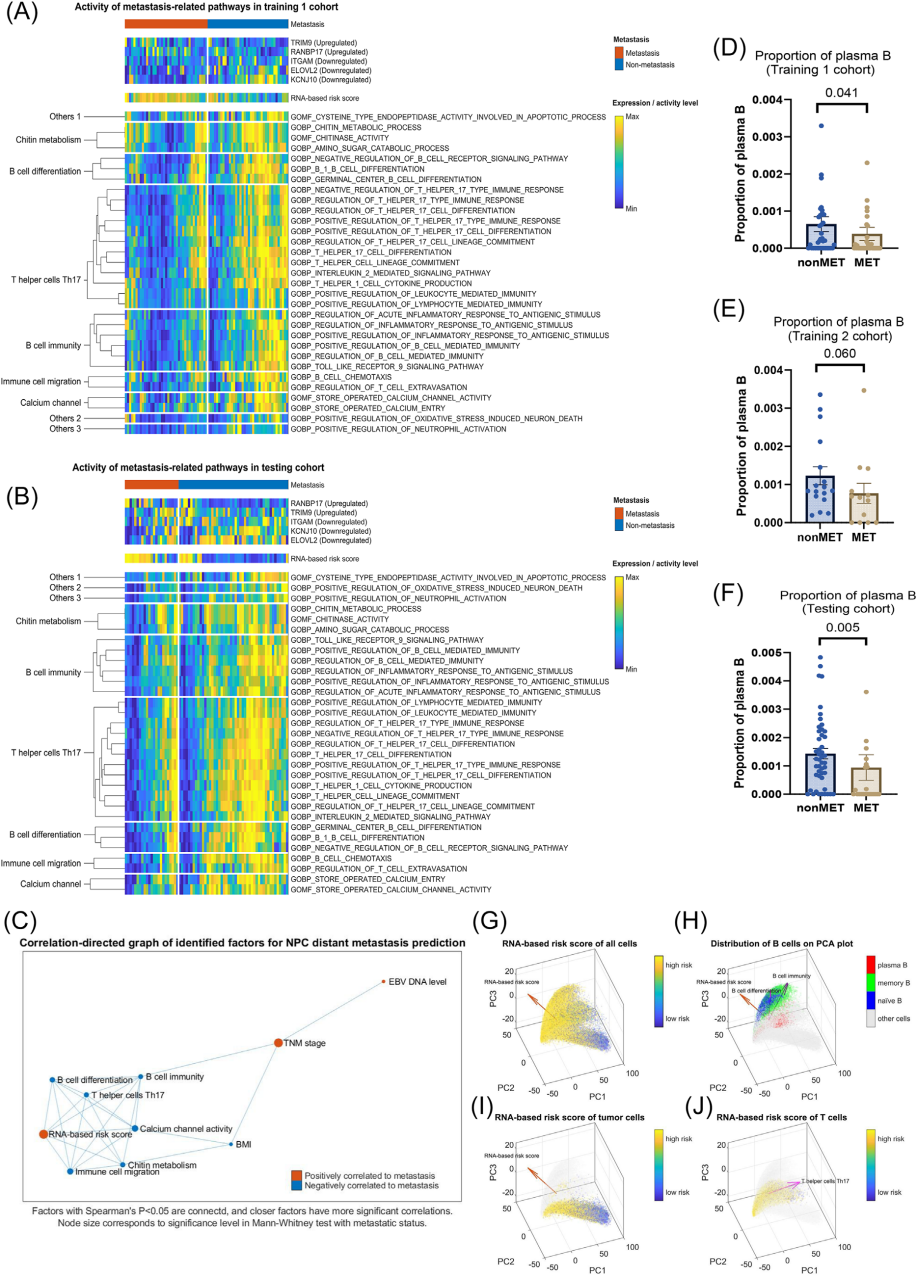
Proposed RNA‐based risk score is negatively correlated to activities of immune functions. (A and B) Thirty‐one pathways showed significantly different pathway activity levels in metastatic nasopharyngeal carcinoma (MET‐NPC) samples (Mann‒Whitney test, *p* < .05) in all the cohorts in this study. (C) Factors with higher significance levels in Spearman's correlation analysis are plotted closer to one another. Size and colour of nodes representing the pathways represent the significance (Mann‒Whitney *p*‐value) and direction of its association with NPC metastasis. (D and F) Proportion of plasma B in biopsy samples from the MET group was significantly lower than the non‐MET group in both training 1 and testing cohorts. (E) A similar trend in proportion difference can be seen in the training 2 cohort, albeit not statistically significant, presumably due to its smaller sample size. (G‒J) Principal component analysis (PCA) plots of single cells from primary tumours, and the linear projection of RNA risk scores and pathway activities on the identified first three principle components that reflect their major varying directions.

To further investigate the functional differences between MET‐NPC and non‐MET‐NPC and identify the biomedical mechanisms underlying the RNA‐based risk score, pathway activities of Gene Ontology Biological Processes and Molecular Function (GOBP/GOMF) gene sets were computed using bulk RNA‐seq data. Thirty‐one pathways with significantly different activities between MET‐NPC and non‐MET‐NPC samples (Mann‒Whitney test, *p* < .05) were identified in all three cohorts and correlated considerably with RNA‐based risk score (Spearman's correlation, *p *< .05, Figure 4A,B).

The clustering analysis of these identified pathways and their corresponding functions suggested that MET‐NPC had significant dysregulation in chitin metabolism, B‐cell immunity and differentiation, T helper 17 (Th17) cell response, immune cell migration and calcium channel transport. These functional activities were highly related to our RNA‐based metastasis risk score while having minimal correlation with TNM stage (Figure 4C). The proposed RNA‐based risk score and TNM stage were the two most effective predictive factors for metastasis; however, they were not significantly correlated. This may explain the enhanced prediction achieved by adding the risk score to the clinical factor‐based prediction model, as the RNA‐based risk score reflects the activity of biological pathways such as immune cell function and calcium channel transportation, which are not significantly correlated with clinical factors.

Further analysis with PCA on three single‐cell sequencing datasets^6^, ^40^, ^41^ supported that the risk would be reduced if naïve B and memory B get activated with more abundant plasma B cells, which is one of the major components of TLS. There was a specific subset of tumour cells with a significantly higher risk score, which may be a highly aggressive cancer clones worthy of further investigation (Figure 4G‒I). The results also indicated that the Th17 cells contributed more towards the reduction of MET risk.

To confirm the association between immunosuppression and MET‐NPC, deconvolution analysis was performed on the bulk RNA‐seq data. In training 1 and testing cohorts, the proportion of plasma B cells in the overall immune cell population was much lower in MET‐NPC than in non‐MET‐NPC, and a similar trend was observed in training 2 cohort (Figure 4D‒F).

**FIGURE 5:**
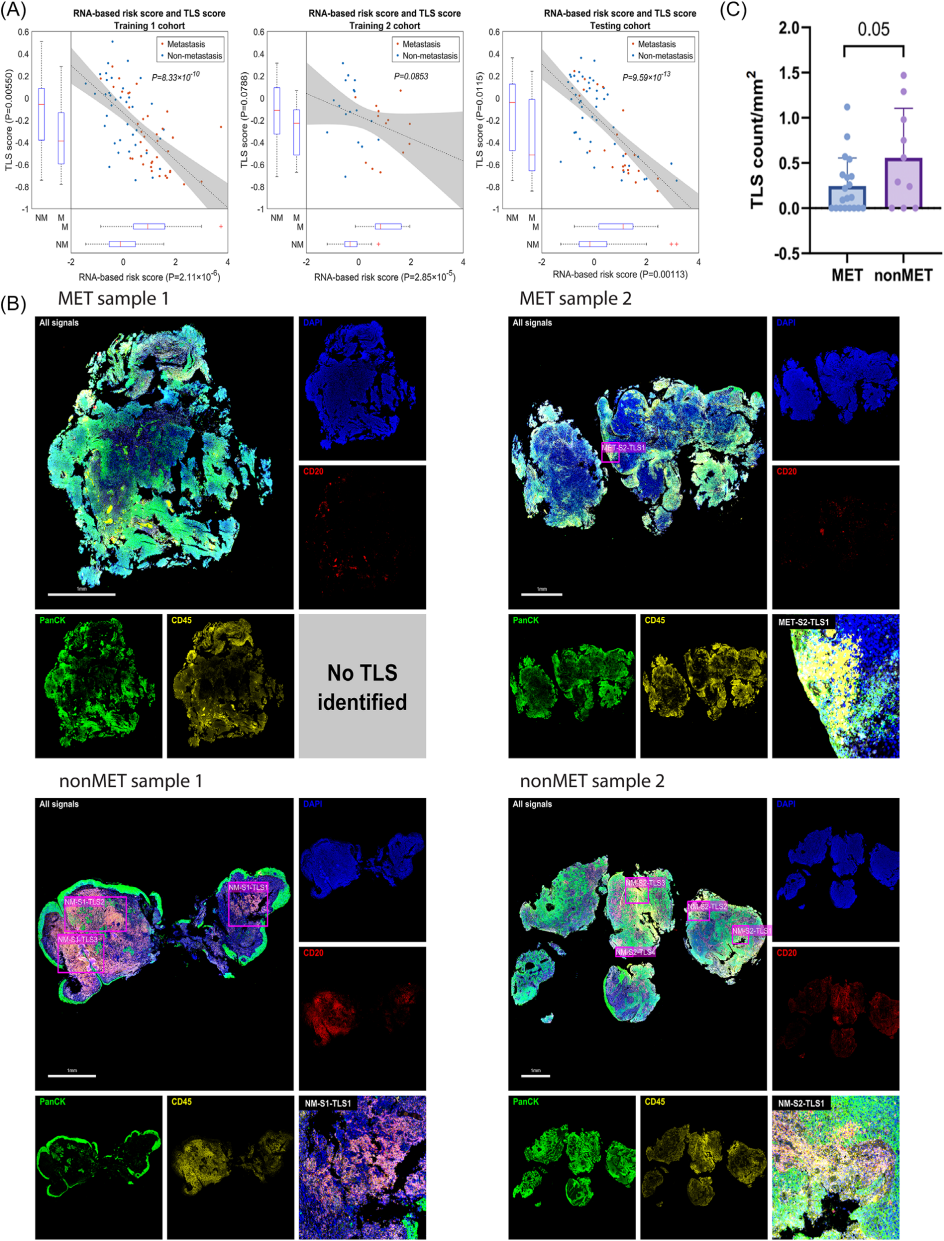
Tertiary lymphoid structures (TLS) are enriched in the non‐metastatic nasopharyngeal carcinoma (non‐MET‐NPC) patients. (A) RNA‐based risk score is negatively correlated to the TLS score (activity score corresponding to the expression of TLS marker genes), and both are significantly related to the metastatic (MET) grouping of NPC patients in training 1 and testing cohorts. (B) According to the biopsy staining from the training 1 cohort, the TLS is significantly enriched in non‐MET‐NPC patients compared to MET patients. (C) Illustration of multiplex staining results for NPC biopsies. Staining signals for PanCK, CD45, CD20 and enlarged TLS regions are presented.

TLSs are indispensable for effective anti‐tumour immunity, as the site of cross‐talk and mutual activation of tumour‐infiltrating lymphocytes, including B cells and Th17 cells.^56^ Therefore, we investigated whether the downregulation of immune‐related pathways and reduced plasma B‐cell populations were linked to potential alterations in TLS formation within MET‐NPC. We estimated a ‘TLS score’ based on the expression level of TLS marker genes^56^ in the literature, whose performance significantly correlated with our RNA‐based risk score in NPC and was significantly downregulated in MET‐NPC (Figure 5A). Further histological analysis of the stained samples from the training 1 cohort (Figure 5B,C) showed that TLS was significantly reduced in the primary tumours of MET‐NPC.

## 4 DISCUSSIONS

Our cross‐cohort analysis of RNA‐seq data identified 29 common gene features associated with NPC distant metastasis, including 15 positive and 14 negative biomarkers. The five‐gene prediction model, developed through Pareto optimisation, demonstrated an AUC of 74.2% on testing cohort. Furthermore, when testing the cohort by combining the gene signature‐based prediction model with clinical information (EBV DNA level, BMI and TMN staging), a notable improvement in prediction accuracy was observed, with the AUC increasing from 84.8% to 90.4%. The combined model is significantly more accurate than both RNA‐only and clinical‐factor‐only models, it suggests that some metastasis‐related factors/mechanisms cannot be effectively reflected by transcriptomics data and a multi‐omics approach may provide a more accurate prediction.

In this study, our proposed multicentre feature selection approach with Pareto optimisation yielded significantly better prediction performances compared to the typical LASSO method with batch effect correction. We have shown an alternative practical approach for selecting predictive features while ensuring robustness across cohorts, however, more comprehensive comparisons are required to evaluate the general advantages of our model.

The identified five genes show significant correlation with TLS markers and there are previous studies that suggest multiple potential mechanisms that contribute to TLS formation or function. For example, *RANBP17* encoding Ran GTP‐binding protein 17, *ELOVL2* encoding elongation of very long chain fatty acids protein 2, and *KCNJ10* encoding ATP‐sensitive inward rectifier potassium channel 10 (also known as Kir4.1), are all involved in regulating B and T‐cell functions.^57^, ^58^, ^59^, ^60^, ^61^ *ITGAM* encoding integrin subunit alpha M (also known as cluster of differentiation molecule 11B CD11b) may influence anti‐tumour immunity by regulating the migration and activation of neutrophils^62^ and macrophages.^63^, ^64^, ^65^ Our analysis further revealed that a subset of cancer cells may have a higher MET potential in NPC patients who lack active immune cell functioning and development, particularly in B and Th17 cells, both of which are relevant to TLS. Correlation analysis between the genes associated with the TLS score and five genes for the risk score further revealed that *RANBP17* is the most significant risk factor, and *KCNJ10* is the most significant protective factor associated with *CXCR5*, *CCL19* and *CCL21* in the bulk tumour, important chemokines that promote TLS formation. Based on the single‐cell sequencing derived from NPC clinical samples, RANBP17 is expressed in EBV‐associated NPC cells, KCNJ10 is detectable in myeloid cells, and *CXCR5* is dormantly expressed by B cells, while cancer‐associated fibroblasts mainly express *CCL19* and *CCL21* (Table S5 and Figures S6 and S7). As a regulator of nucleocytoplasmic transport, RANBP17 binds to the small GTPase Ran, facilitating the movement of molecular cargo. This role is essential for modulating key cellular activities, including proliferation and differentiation. We speculate that RANBP17 may suppress stromal CXCL13, CCL19 and CCL21 through exosome‐mediated mechanisms by reprogramming the cargo of cancer‐derived exosomes to include regulatory RNAs and proteins. Notably, circRANBP17, a circular RNA derived from RANBP17, has been shown to promote tumour progression via miRNA sponging mechanisms,^66^ and RANBP17 itself has been implicated in nuclear transport and immune modulation associated with miRNAs in glioblastoma.^59^ These findings support the plausibility of RANBP17‐driven exosomal remodelling as a mechanism for chemokine suppression in the tumour microenvironment. This process may be inhibited by the myeloid cells expressing KCNJ10, controlling potassium (K⁺) flux and membrane potential, which is not well characterised yet. Together, these mechanisms contribute to a tumour microenvironment that impairs immune cell recruitment and TLS formation. Further systematic investigation is warranted to validate this hypothesis.

NPC is an ‘immune‐hot’ cancer with high lymphocyte infiltration. However, multiple single‐cell RNA‐seq studies have suggested that NPC may possess a highly immunosuppressive tumour microenvironment.^40^, ^67^ The five marker genes in our RNA risk score were characterised in other studies for their involvement in cancer metastasis (Table S4) and immune cell functions. TLS are ectopic lymphoid organs vital for B and T‐cell‐mediated anti‐tumour immune responses,^68^ where B cells are activated to differentiate into plasma B cells by undergoing class‐switching and somatic hypermutation, often with the assistance of follicular helper T cells and follicular dendritic cells.^69^ Consistent with the association between plasma B‐cell‐abundant mature TLS and good clinical outcomes in multiple cancers, such as non‐small‐cell lung cancer and pancreatic cancer,^70^, ^71^, ^72^ we identified positive correlations of TLS score within non‐MET and a striking reduction of plasma B cell in MET‐NPC. However, the biological mechanisms underlying reduced TLS formation and plasma B cells in MET‐NPC remain unclear. Tumour‐infiltrating Th17 cells are involved in TLS formation^73^ and as a component of TLS, may directly stimulate B‐cell differentiation, proliferation and antibody production by secreting cytokines such as IL‐17 and IL‐21.^74^, ^75^ As we found that Th17‐related pathways were downregulated in MET‐NPC, we speculate that interactions between Th17 and B cells within TLS may be inhibited, preventing the differentiation of B cells into plasma B cells. Another possibility is that cancer cells with high MET potential form the pro‐MET niches, which inhibit B‐cell differentiation and survival. Further studies are needed to elucidate the interactions between Th17 and B cells, and malignant cancer cells in NPC metastasis.

Regarding the integration of the proposed risk score with the existing clinical diagnostic and treatment workflow, the score can be evaluated before initiating first‐line treatment. For patients with low risk of metastases, the radiation dose can be controlled to reduce long‐term damage. For those with high risk, a more aggressive approach can be taken, involving the addition of induction chemotherapy or a three‐drug regimen, to prevent metastasis. In addition, further investigation of the detailed mechanisms underlying the TLS formation may help to identify the treatment strategy and therapeutic targets to further boost anti‐tumour immunity. It can thus be concluded that the score in question has the potential to contribute to the field of precision medicine for patients suffering from NPC, with the possibility of benefit to both the MET and non‐MET groups.

In conclusion, we developed an RNA‐based risk score for MET‐NPC risk prediction, which could yield significantly improved prediction accuracy when combined with clinical information‐based predictive models. Our findings revealed that downregulation of anti‐tumour immunity, particularly B‐cell immunity and TLS formation, may be associated with NPC metastasis, providing insight into the functional differences between MET‐NPC and non‐MET‐NPC tumours. Furthermore, the multi‐cohort Pareto‐optimisation‐based feature selection approach offers a practical method to explicitly avoid model overfitting and achieve a more robust model, which can be applied to a broader range of studies.

## AUTHOR CONTRIBUTIONS

Wei Dai and Yunfei Xia designed and supervised this study. Zhaozheng Hou developed the study methodology. Zhaozheng Hou, Ping Feng, Chi‐Leung Chiang, Kazi Anisha Isla, Songran Liu, Ying Wang, Zilu Huang, Victor Ho‐Fun Lee, Anne Wing‐Mui Lee, Dora Lai‐Wan Kwong, Jason Wing Hon Wong, Wai Tong Ng, Wei Dai and Yunfei Xia contributed to the sample and data acquisition. Zhaozheng Hou, Kazi Anisha Islam, Songran Liu, Yingpei Zhang, Michael King‐Yung Chung, Wei Dai and Yunfei Xia contributed to the data analysis and interpretation. Wei Dai and Yunfei Xia acquired the funding for this project. All the authors contributed to writing, reviewing and revising the manuscript.

## CONFLICT OF INTEREST STATEMENT

The authors declare they have no conflicts of interest.

## ETHICS STATMENT

This study was approved by the Institutional Review Board of the University of Hong Kong for training 1 cohort (UW 09‐125), and by the Ethics Committee of Sun Yat‐Sen University Cancer Center for training 2 and testing cohorts (G2021‐110‐01).

## Supporting information



Supporting Information

## Data Availability

The raw sequence data reported in this paper have been deposited in the Genome Sequence Archive^76^ in National Genomics Data Center,^77^ China National Center for Bioinformation/Beijing Institute of Genomics, Chinese Academy of Sciences (RRID:SCR_025826, GSA‐Human: HRA008959) that are publicly accessible at https://ngdc.cncb.ac.cn/gsa‐human.
