# Supplementary material for: Tertiary lymphoid structure‐related RNA indicator as metastasis risk factor in nasopharyngeal carcinoma
Source: Clin Transl Med. 2025 Dec 15;15(12):e70539. doi: 10.1002/ctm2.70539 (PMC12705341; doi:10.1002/ctm2.70539)
Supplement: Supplementary file 1 — Supporting Information [file CTM2-15-e70539-s001.docx]

# Multicenter Study to Establish RNA-based Risk Model for Nasopharyngeal Carcinoma Distant Metastasis

Zhaozheng Hou, PhD^1^; Ping Feng, PhD^2^; Kazi Anisha Islam, BEng^1^; Songran Liu, PhD^1,3,4^; Ying Wang, PhD^2^; Yingpei Zhang, MSc^1^; Michael King-Yung Chung, BSc^1^; Ngar-Woon Kam, PhD^5^; Zilu Huang, PhD^2^; Victor Ho-Fun Lee, MD^1,6^; Anne Wing-Mui Lee, MD ^1,6^; Dora Lai-Wan Kwong, MD ^1,6^; Wai Tong Ng, MD ^1,6^; Yunfei Xia, MD ^2,3^; Wei Dai*, PhD ^1,6^

1. Department of Clinical Oncology, University of Hong Kong, Hong Kong (SAR), PR China.
2. Department of Radiation Oncology, Sun Yat-sen University Cancer Centre, Guangzhou, PR China
3. State Key Laboratory of Oncology in South China, Collaborative Innovation Center for Cancer Medicine, Guangdong Key Laboratory of Nasopharyngeal Carcinoma Diagnosis and Therapy, Sun Yat-sen University Cancer Center, Guangzhou, PR China
4. Department of Pathology, Sun Yat-sen University Cancer Centre, Guangzhou, PR China
5. Laboratory for Synthetic Chemistry and Chemical Biology, Hong Kong (SAR), PR China
6. Department of Clinical Oncology, Shenzhen Key Laboratory for cancer metastasis and personalized therapy, The University of Hong Kong-Shenzhen Hospital, Shenzhen, PR China.

##
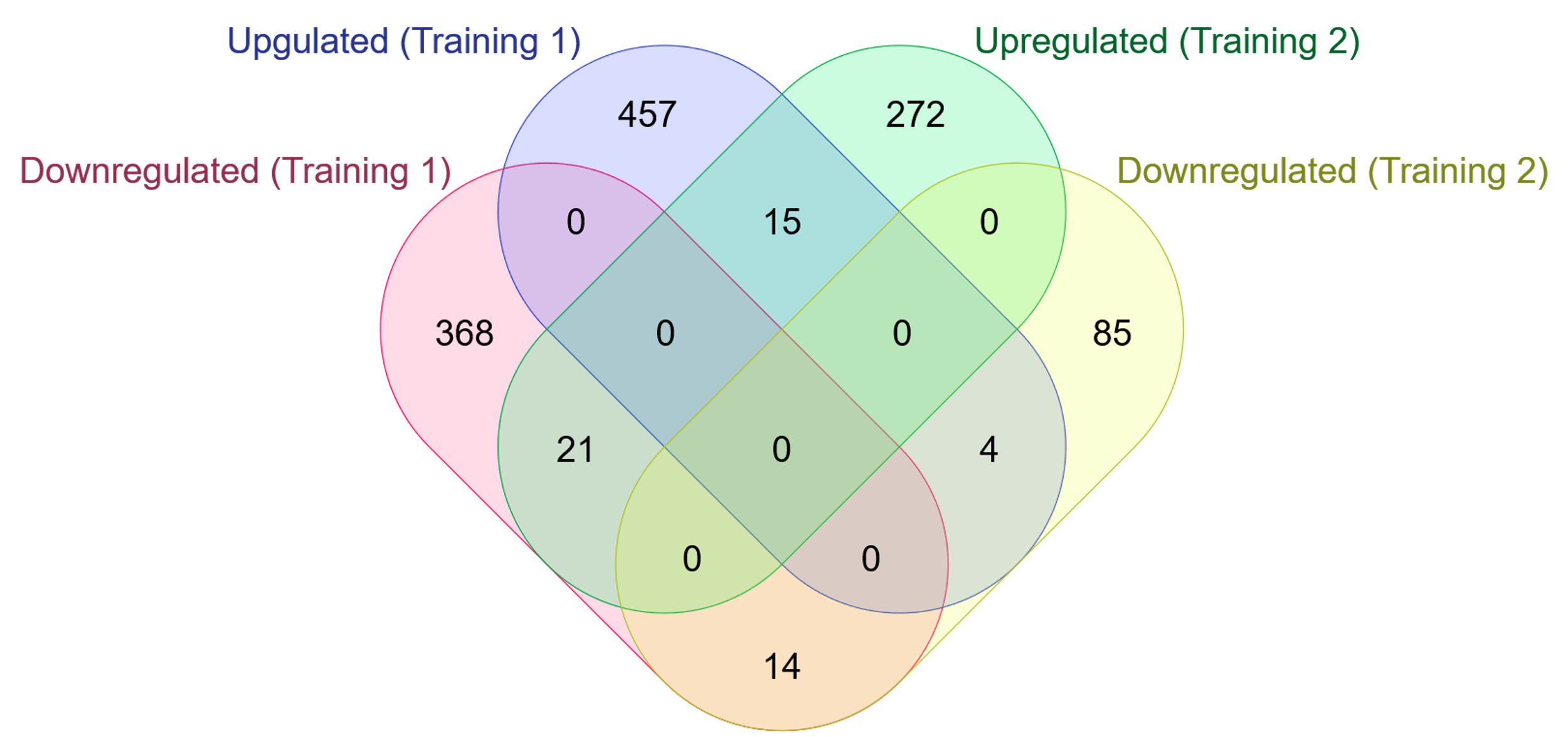
Supplement figures and tables

**Figure S1. Number of gene markers identified from training 1 and training 2 cohorts.** 29 genes were significantly up/downregulated in NPC with distant metastasis in both training cohorts.


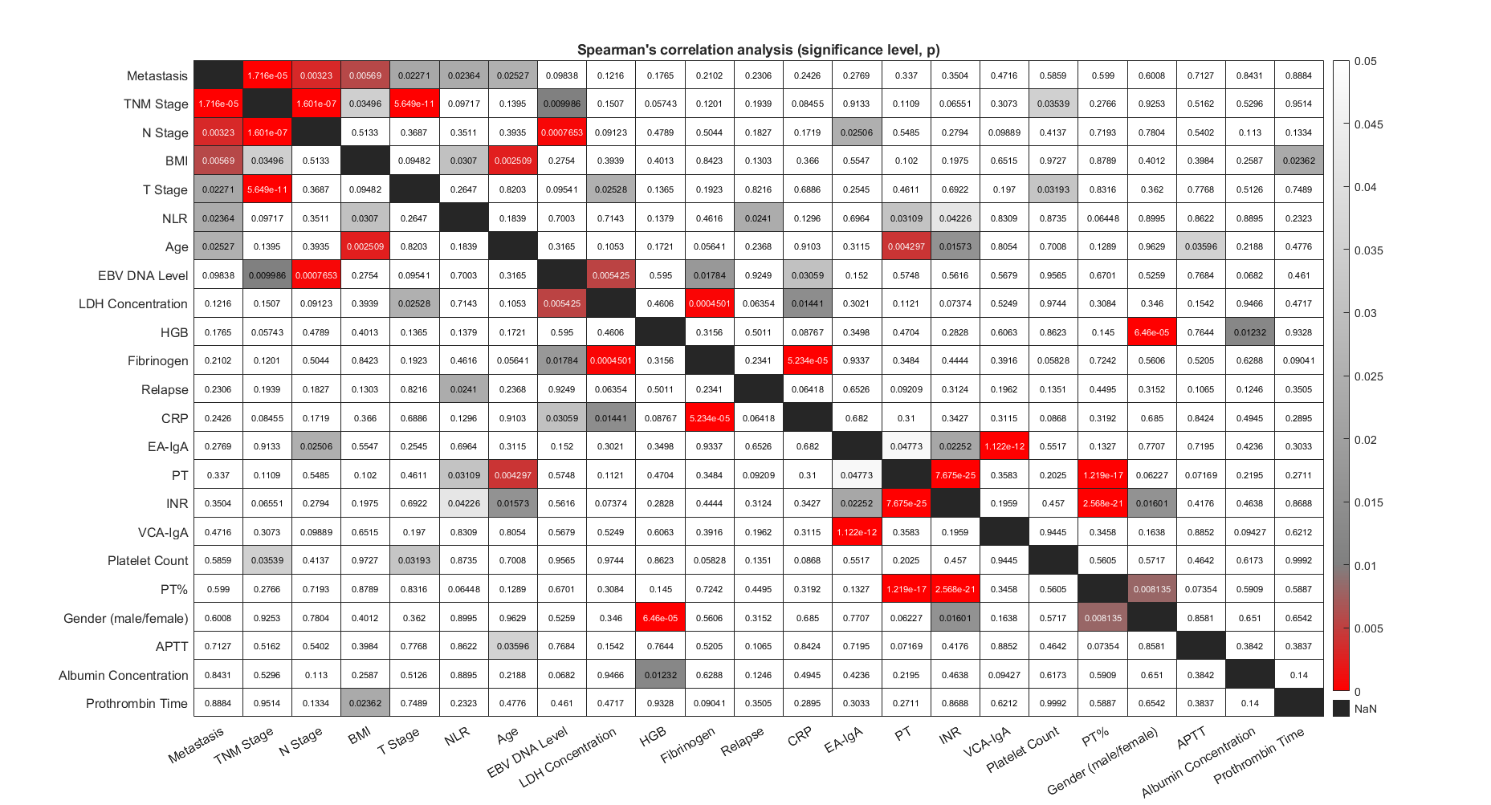


**Figure S2. The significance level of Spearman’s correlation for clinical parameters.**


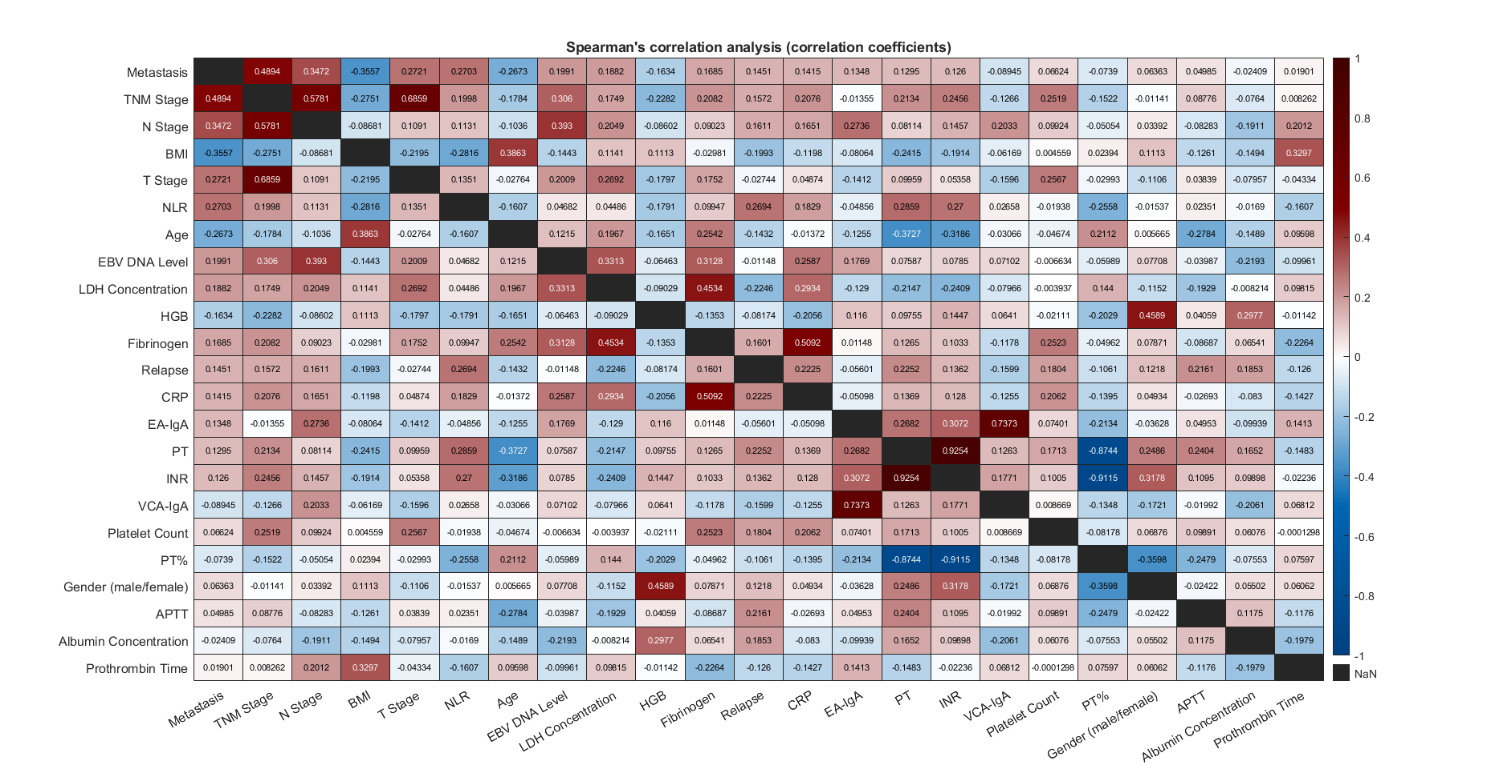
**Figure S3. The correlation coefficient of Spearman’s correlation for clinical parameters.**


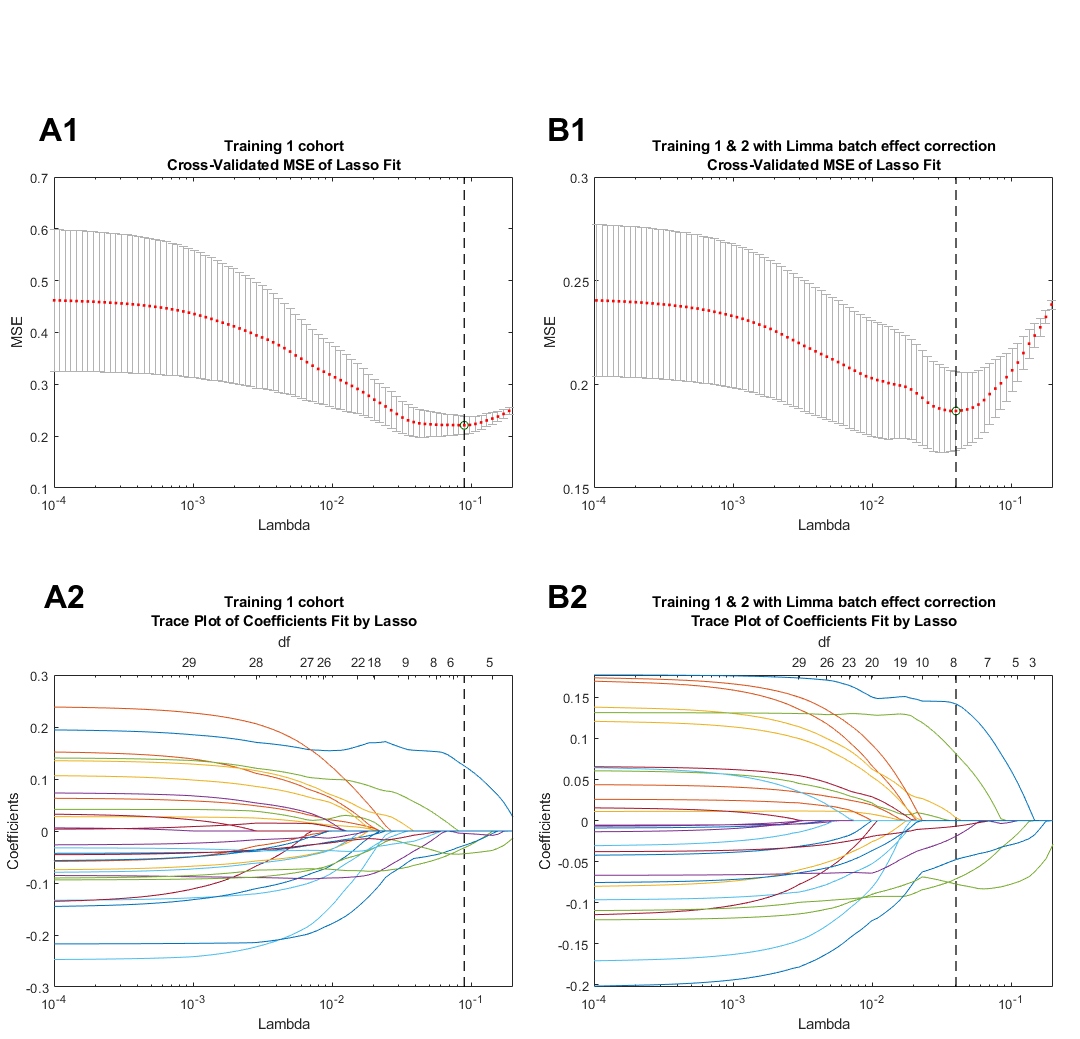
**Figure S4. Detailed results of the Lasso feature selection on training cohort 1 and the combination of 2 training cohorts.** (**A**) In the analysis only based on training cohort 1, the best predictive model corresponds to a five-gene combination. (**B**) In the analysis based on combining training cohorts 1 and 2 with batch effect correction, the best predictive model corresponds to an eight-gene combination.


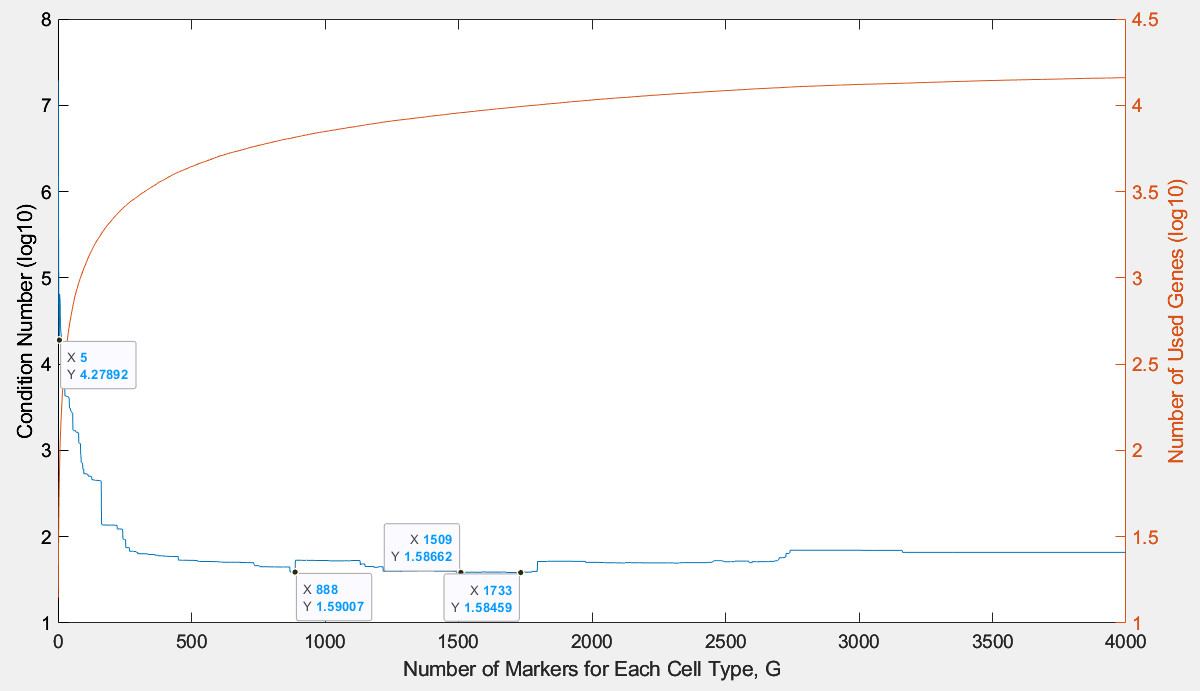
**Figure S5. Condition number of the matrices with different G values.** The labeled points indicate locally optimal solutions that minimize the number of genes without increasing the condition number of the signature matrix. A G value of 1509 was selected as it leads to the best-fitting results in terms of fitting the remaining residual of gene expression.


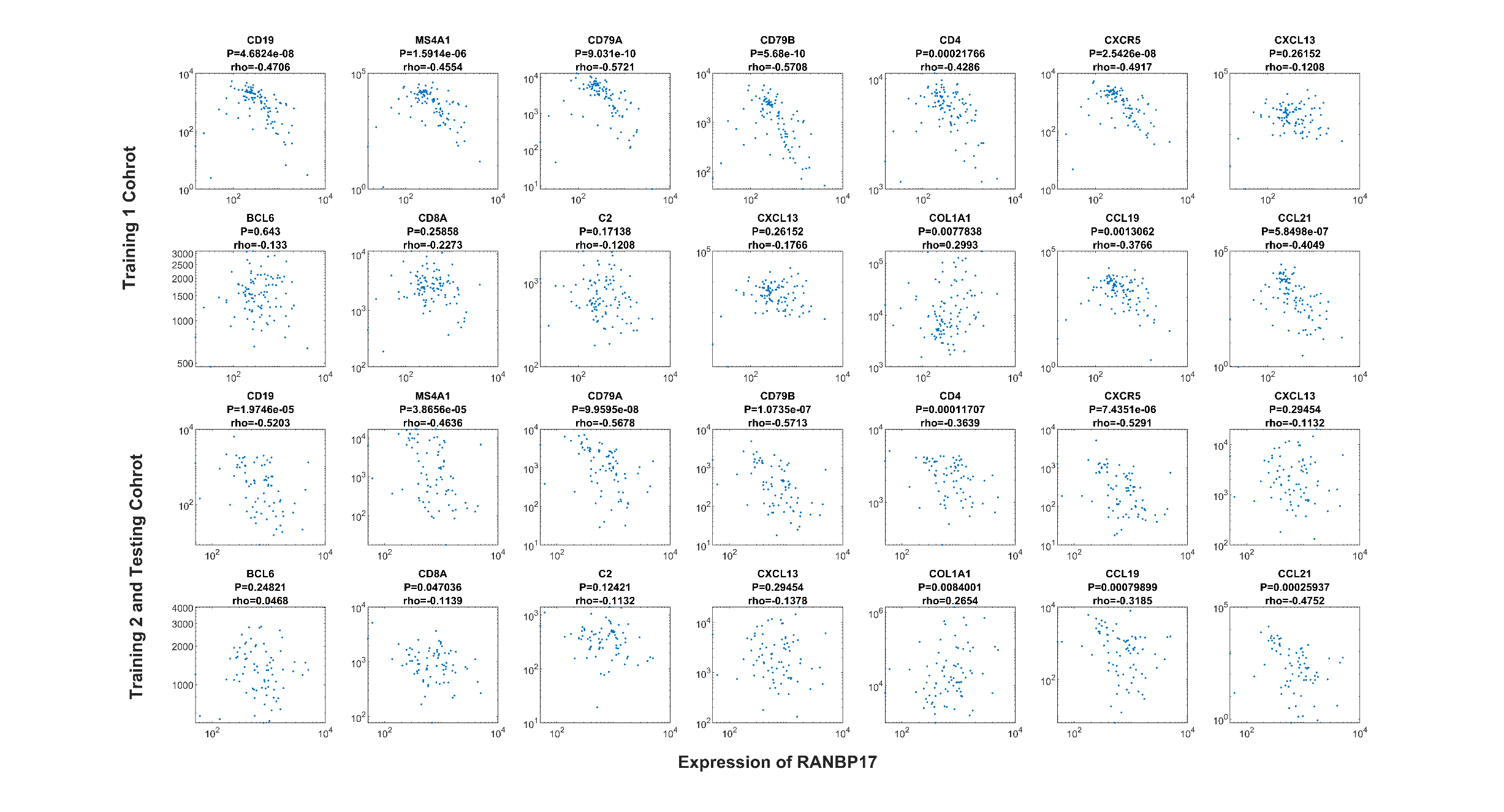
**Figure S6.** **Spearman correlation between RANBP17 and TLS markers.**


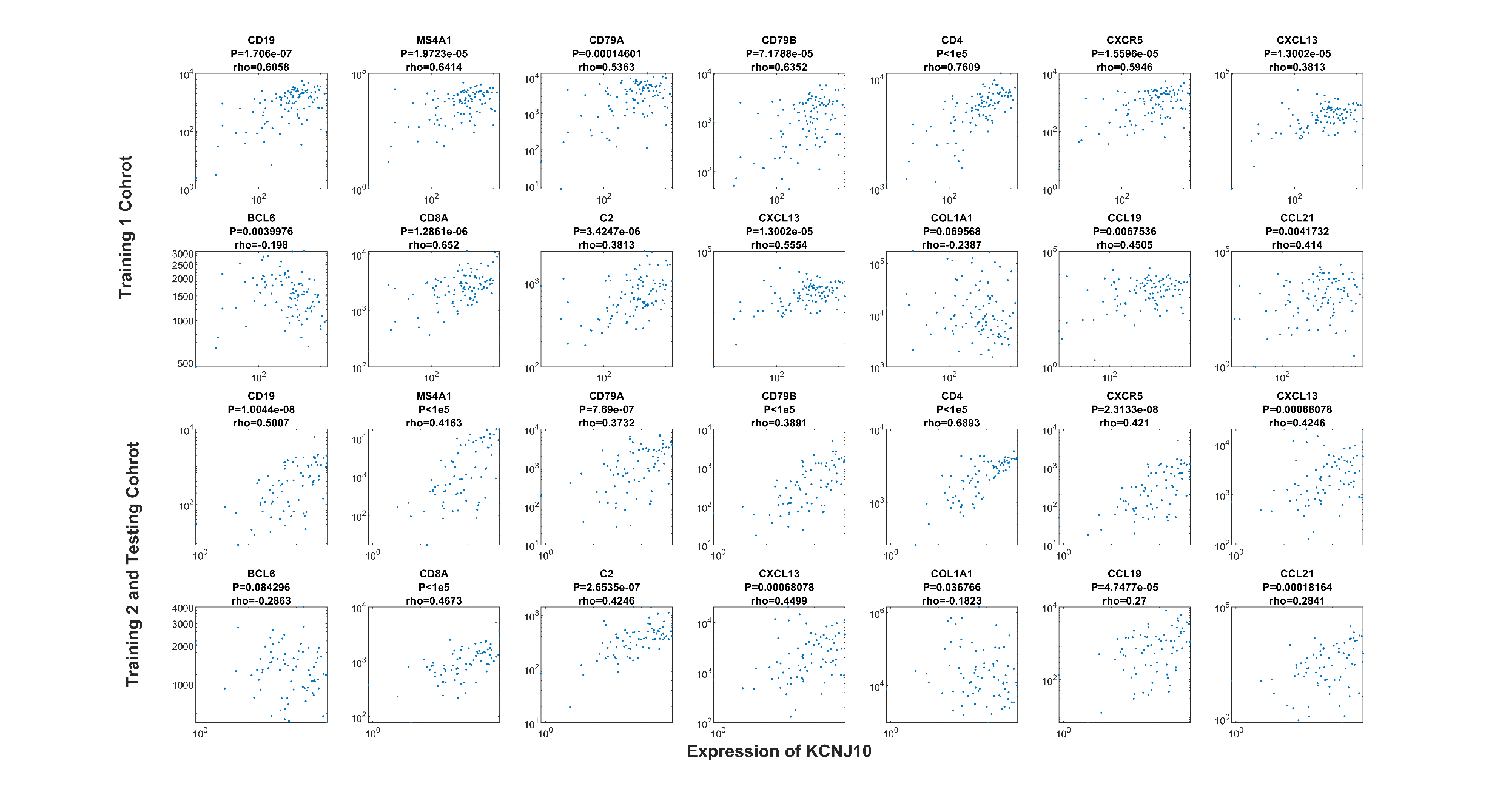
**Figure S7.** **Spearman correlation between KCNJ10 and TLS markers.**

**Figure S8.** **Comparison of CIBERSORT deconvolution results (y-axis) and the results from the “expression-variance” approach (x-axis).**


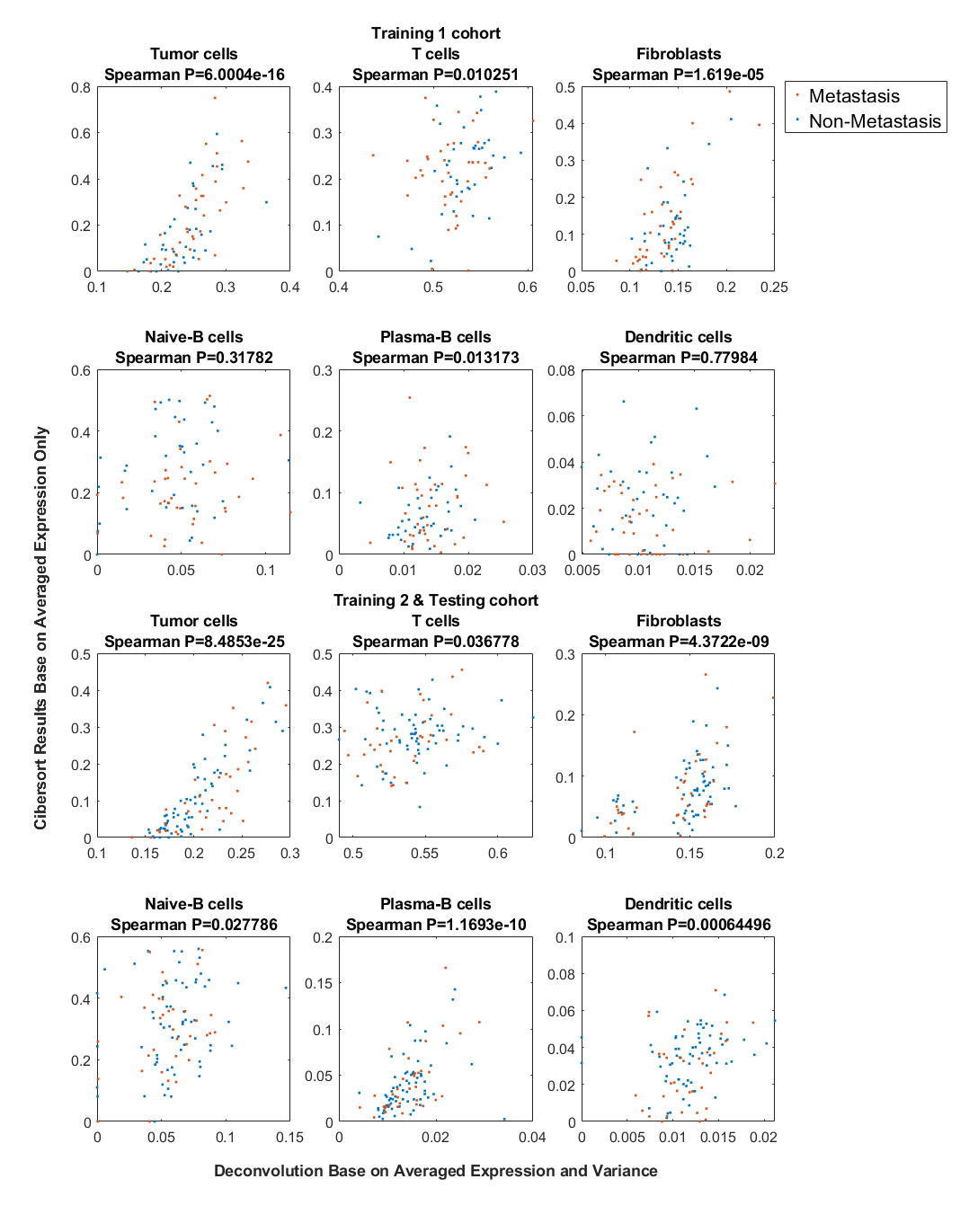


**
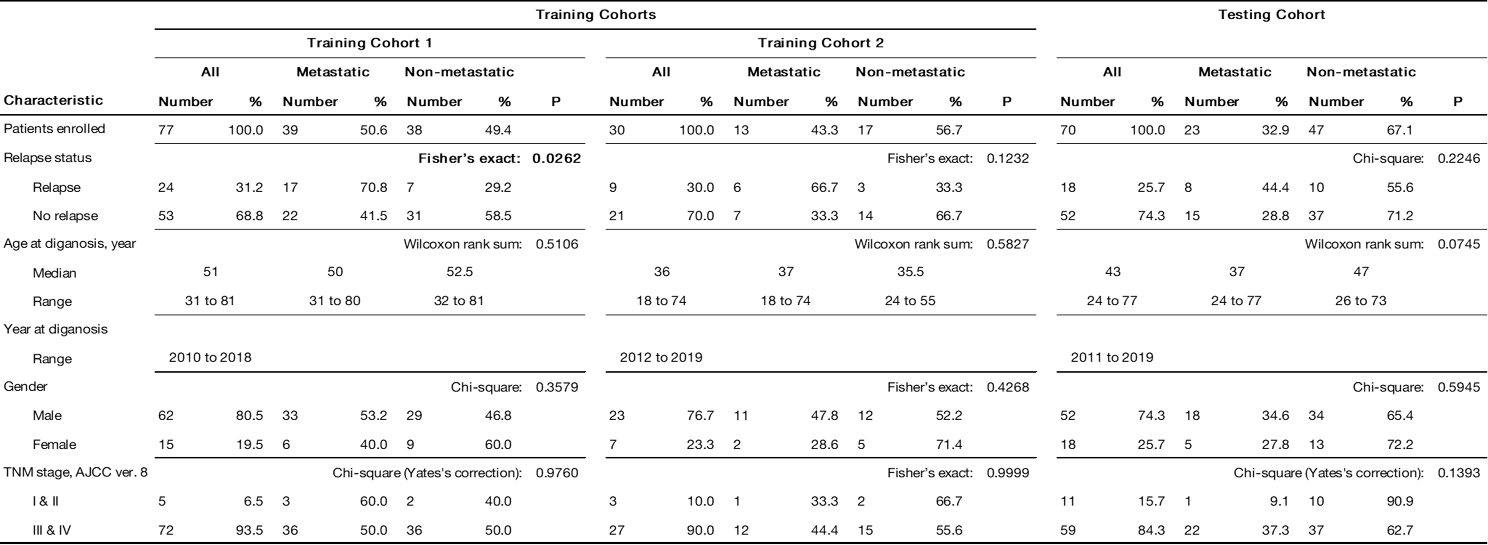
Table S1. Clinical characteristics in association with metastasis for the involved cohorts in this study.**

**Table S2. Table of available clinical characteristics in the testing cohort.**

**Table S3. Results of multi-variable analysis of clinical characteristics with significant correlation to distant metastasis.**

**Table S4. The contribution of the five selected genes in cancer metastasis from other studies.**

| Related gene | Pathway | Previous study | Cancer type | Type |
| --- | --- | --- | --- | --- |
| RANBP17, M+ | RANBP17 | 2021, (1) | NPC | Experiment-based |
|  |  | 2014, (2)  2022, (3) | HNSCC | Experiment-based |
|  | GOBP_POSITIVE_REGULATION_OF_HIPPO_SIGNALING | 2016, (4)  2023, (5) | Multiple cancer types | Review |
|  | GOBP_REGULATION_OF_CALCIUM_ION_TRANSMEMBRANE_TRANSPORT | 2018, (6)  2021, (7) | Multiple cancer types (including NPC) | Review |
|  |  | 2015, (8) | NPC | Statistical study |
|  | GOBP_PYRIDINE_CONTAINING_COMPOUND_CATABOLIC_PROCESS | 2020, (9)  2021, (10) | Multiple cancer types | Review |
| TRIM9, M+ | TRIM9 | 2020, (11) | uterine leiomyoma (UM) | Experiment-based |
|  | GOBP_LINOLEIC_ACID_METABOLIC_PROCESS | 2024, (12)  2024, (13) | Multiple cancer types | Review |
| ITGAM, M- | ITGAM | 2020, (14) | Esophageal cancer | Statistical study |
|  | GOBP_REGULATION_OF_ENDOTHELIAL_CELL_DEVELOPMENT | 2023, (15) | Multiple caner types | Review |
|  | GOBP_REGULATION_OF_FEVER_GENERATION |  |  |  |
|  | GOBP_NEGATIVE_REGULATION_OF_CELLULAR_AMINE_METABOLIC_PROCESS | 2023, (16) | Multiple caner types | Review |
| ELOVL2, M- | ELOVL2 | 2019, (17) | breast cancer | Experiment-based |
|  | GOBP_RESPONSE_TO_WATER_DEPRIVATION |  |  |  |
|  | GOBP_POSITIVE_REGULATION_OF_ENDOTHELIAL_CELL_APOPTOTIC_PROCESS |  |  |  |
| KCNJ10, M- | KCNJ10 | 2020, (18)  2022, (19)  2023, (20) | Multiple caner types | Review |
|  | GOCC_GUANYL_NUCLEOTIDE_EXCHANGE_FACTOR_COMPLEX | 2023, (21) | Multiple caner types | Review |
|  | GOBP_REGULATION_OF_ENDOSOME_TO_PLASMA_MEMBRANE_PROTEIN_TRANSPORT | 2024, (22) | HeLa | Experiment-based |
|  | GOBP_NEGATIVE_REGULATION_OF_MYOSIN_LIGHT_CHAIN_PHOSPHATASE_ACTIVITY | 2021, (23) | LLC | Experiment-based |
|  |  | 2023, (24) | PDAC | Experiment-based |
|  | GOBP_REVERSE_CHOLESTEROL_TRANSPORT | 2023, (25) | LLC | Experiment-based |
|  |  | 2023, (26) | Multiple caner types | Review |
|  | GOCC_SHELTERIN_COMPLEX | 2021, (27)  2024, (28) | Multiple caner types | Review |
|  | GOBP_L_GLUTAMATE_IMPORT_ACROSS_PLASMA_MEMBRANE | 2023, (29) | Multiple caner types | Review |


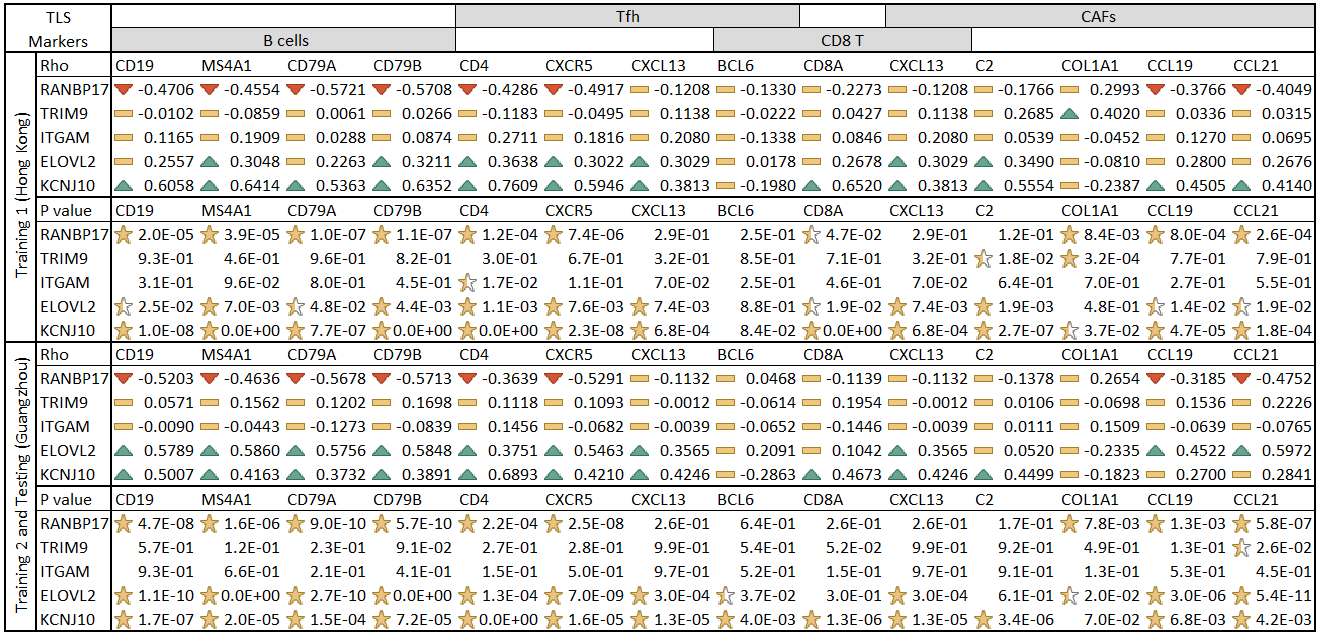
**Table S5.** Spearman correlation analysis of the expression level of identified marker genes with TLS markers.


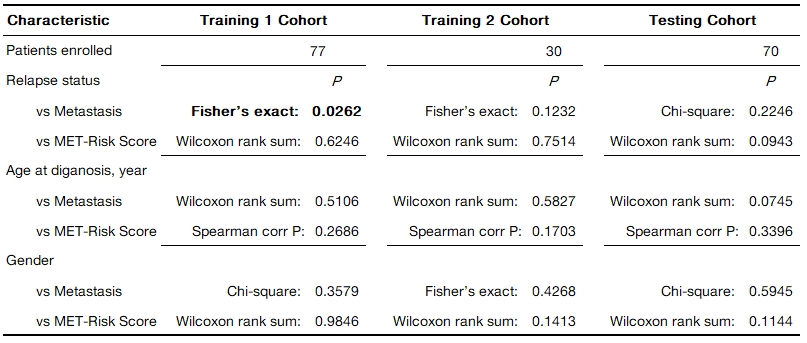
**Table S6.** Metastasis proportion and RNA risk score in different cohorts and patient subgroups.

## Reference

1. Zhou M, Zhang P, Zhao Y, Liu R, Zhang Y. Overexpressed circRANBP17 acts as an oncogene to facilitate nasopharyngeal carcinoma via the miR-635/RUNX2 axis. Journal of Cancer **2021**;12(14):4322.

2. Tonigold M, Rossmann A, Meinold M, Bette M, Märken M, Henkenius K*, et al.* A cisplatin-resistant head and neck cancer cell line with cytoplasmic p53 mut exhibits ATP-binding cassette transporter upregulation and high glutathione levels. Journal of cancer research and clinical oncology **2014**;140:1689-704.

3. Mandic R, Marquardt A, Terhorst P, Ali U, Nowak-Rossmann A, Cai C*, et al.* The importin beta superfamily member RanBP17 exhibits a role in cell proliferation and is associated with improved survival of patients with HPV+ HNSCC. BMC cancer **2022**;22(1):785.

4. van Rensburg HJJ, Yang X. The roles of the Hippo pathway in cancer metastasis. Cellular signalling **2016**;28(11):1761-72.

5. Zhou T, Li X, Liu J, Hao J. The Hippo/YAP signaling pathway: the driver of cancer metastasis. Cancer Biology & Medicine **2023**;20(7):483.

6. Bong AH, Monteith GR. Calcium signaling and the therapeutic targeting of cancer cells. Biochimica et Biophysica Acta (BBA)-Molecular Cell Research **2018**;1865(11):1786-94.

7. Cui C, Zhang Y, Liu G, Zhang S, Zhang J, Wang X. Advances in the study of cancer metastasis and calcium signaling as potential therapeutic targets. Exploration of Targeted Anti-tumor Therapy **2021**;2(3):266.

8. Chen J-P, Wang J, Luan Y, Wang C-X, Li W-H, Zhang J-B*, et al.* TRPM7 promotes the metastatic process in human nasopharyngeal carcinoma. Cancer letters **2015**;356(2):483-90.

9. Mollick T, Laín S. Modulating pyrimidine ribonucleotide levels for the treatment of cancer. Cancer & metabolism **2020**;8(1):12.

10. Wang W, Cui J, Ma H, Lu W, Huang J. Targeting pyrimidine metabolism in the era of precision cancer medicine. Frontiers in oncology **2021**;11:684961.

11. Yang F, Liu H, Yu Y, Xu L. TRIM9 overexpression promotes uterine leiomyoma cell proliferation and inhibits cell apoptosis via NF-κB signaling pathway. Life Sciences **2020**;257:118101.

12. Vogel FC, Chaves-Filho AB, Schulze A. Lipids as mediators of cancer progression and metastasis. Nature Cancer **2024**;5(1):16-29.

13. Terry AR, Hay N. Emerging targets in lipid metabolism for cancer therapy. Trends in Pharmacological Sciences **2024**.

14. Zhou M-H, Wang X-K. Microenvironment-related prognostic genes in esophageal cancer. Translational cancer research **2020**;9(12):7531.

15. Yao X, Zeng Y. Tumour associated endothelial cells: origin, characteristics and role in metastasis and anti-angiogenic resistance. Frontiers in Physiology **2023**;14:1199225.

16. You M, Xie Z, Zhang N, Zhang Y, Xiao D, Liu S*, et al.* Signaling pathways in cancer metabolism: mechanisms and therapeutic targets. Signal Transduction and Targeted Therapy **2023**;8(1):196.

17. Kang YP, Yoon J-H, Long NP, Koo G-B, Noh H-J, Oh S-J*, et al.* Spheroid-induced epithelial-mesenchymal transition provokes global alterations of breast cancer lipidome: a multi-layered omics analysis. Frontiers in oncology **2019**;9:145.

18. Fan JJ, Huang X. Ion channels in cancer: Orchestrators of electrical signaling and cellular crosstalk. Targets of Cancer Diagnosis and Treatment: Ion Transport in Tumor Biology **2020**:103-33.

19. Zúñiga L, Cayo A, González W, Vilos C, Zúñiga R. Potassium channels as a target for cancer therapy: Current perspectives. OncoTargets and therapy **2022**;15:783.

20. Li M, Tian P, Zhao Q, Ma X, Zhang Y. Potassium channels: Novel targets for tumor diagnosis and chemoresistance. Frontiers in Oncology **2023**;12:1074469.

21. Cervantes-Villagrana RD, García-Jiménez I, Vázquez-Prado J. Guanine nucleotide exchange factors for Rho GTPases (RhoGEFs) as oncogenic effectors and strategic therapeutic targets in metastatic cancer. Cellular Signalling **2023**;109:110749.

22. Wenzel EM, Pedersen NM, Elfmark LA, Wang L, Kjos I, Stang E*, et al.* Intercellular transfer of cancer cell invasiveness via endosome-mediated protease shedding. Nature Communications **2024**;15(1):1277.

23. Kim Y-E, Gwak S-H, Hong B-J, Oh J-M, Choi H-S, Kim MS*, et al.* Effects of ultra-high doserate FLASH irradiation on the tumor microenvironment in Lewis lung carcinoma: Role of myosin light chain. International Journal of Radiation Oncology* Biology* Physics **2021**;109(5):1440-53.

24. Samain R, Maiques O, Monger J, Lam H, Candido J, George S*, et al.* CD73 controls Myosin II–driven invasion, metastasis, and immunosuppression in amoeboid pancreatic cancer cells. Science advances **2023**;9(42):eadi0244.

25. Raccosta L, Marinozzi M, Costantini S, Maggioni D, Ferreira LM, Corna G*, et al.* Harnessing the reverse cholesterol transport pathway to favor differentiation of monocyte-derived APCs and antitumor responses. Cell Death & Disease **2023**;14(2):129.

26. Liang S, Tian S, Kang X, Zhang D, Wu W, Yu L. Skin lesion classification base on multi-hierarchy contrastive learning with pareto optimality. Biomedical Signal Processing and Control **2023**;86:105187.

27. Khodadadi E, Mir SM, Memar MY, Sadeghi H, Kashiri M, Faeghiniya M*, et al.* Shelterin complex at telomeres: roles in cancers. Gene Reports **2021**;23:101174.

28. Brankiewicz W, Kallingal A, Krzemieniecki R, Baginski M. Targeting shelterin proteins for cancer therapy. Drug Discovery Today **2024**:104056.

29. Jin J, Byun J-K, Choi Y-K, Park K-G. Targeting glutamine metabolism as a therapeutic strategy for cancer. Experimental & Molecular Medicine **2023**;55(4):706-15.
